# CstF-64 supports pluripotency and regulates cell cycle progression in embryonic stem cells through histone 3′ end processing

**DOI:** 10.1093/nar/gku551

**Published:** 2014-06-21

**Authors:** Bradford A. Youngblood, Petar N. Grozdanov, Clinton C. MacDonald

**Affiliations:** Department of Cell Biology & Biochemistry, Texas Tech University Health Sciences Center, 3601 4th Street, Lubbock, TX 79430-6540, USA

## Abstract

Embryonic stem cells (ESCs) exhibit a unique cell cycle with a shortened G_1_ phase that supports their pluripotency, while apparently buffering them against pro-differentiation stimuli. In ESCs, expression of replication-dependent histones is a main component of this abbreviated G_1_ phase, although the details of this mechanism are not well understood. Similarly, the role of 3′ end processing in regulation of ESC pluripotency and cell cycle is poorly understood. To better understand these processes, we examined mouse ESCs that lack the 3′ end-processing factor CstF-64. These ESCs display slower growth, loss of pluripotency and a lengthened G_1_ phase, correlating with increased polyadenylation of histone mRNAs. Interestingly, these ESCs also express the τCstF-64 paralog of CstF-64. However, τCstF-64 only partially compensates for lost CstF-64 function, despite being recruited to the histone mRNA 3′ end-processing complex. Reduction of τCstF-64 in CstF-64-deficient ESCs results in even greater levels of histone mRNA polyadenylation, suggesting that both CstF-64 and τCstF-64 function to inhibit polyadenylation of histone mRNAs. These results suggest that CstF-64 plays a key role in modulating the cell cycle in ESCs while simultaneously controlling histone mRNA 3′ end processing.

## INTRODUCTION

Although embryonic stem cells (ESCs) are defined by their pluripotent and self-renewal properties, little is known about how they control their cell cycle. The ESC cell cycle is abbreviated, with a shortened G_1_ phase and a high proportion of cells in S phase. The abbreviated G_1_ phase is thought to protect ESCs from pro-differentiation signals that disrupt the stem cell state, suggesting that pluripotency and self-renewal are intimately linked to the cell cycle (1–5). However, unlike somatic cells, ESCs do not display the typical growth factor-dependent restriction (R) point and functional Rb-E2F pathways that ensures competency for DNA replication in G_1_ phase. Instead, synthesis of replication-dependent histones controls the G_1_/S phase transition in ESCs by a mechanism that is not yet understood (4,6–9).

Replication-dependent histone mRNAs, unlike most eukaryotic mRNAs, are not usually processed with a 3′ poly(A) tail. Instead, the majority of these transcripts end in a conserved stem loop that allows for their precise cell cycle regulation (10,11), although in growing cells, some histone mRNAs end in poly(A) tails (12,13). Histone mRNA 3′ end processing is carried out by a subset of specialized ribonucleoproteins that recognize specific elements on the nascent histone mRNA. A unique U7 small ribonucleoprotein (U7 snRNP) complex base pairs with a purine-rich histone mRNA downstream element (HDE). The U7 snRNP recruits FLICE-associated huge protein, FLASH (14) and other proteins, including many that have overlapping roles in polyadenylation: CPSF-73 and CPSF-100, CPSF-160, CFI_m_68, symplekin and Fip1 (15–18).

Recently, it was discovered that CstF-64 was also part of the replication-dependent histone mRNA 3′ end-processing complex (15,18,19). CstF-64 (gene symbol *Cstf2*) is the RNA-binding component of the cleavage stimulation factor that is required for mRNA polyadenylation (20). A paralog of CstF-64, τCstF-64 (gene symbol *Cstf2t*) is expressed in testis and brain (21–24) and is required for spermatogenesis (25–27). Expression of CstF-64 increases in embryonic stem cells and in the reprogramming of somatic cells into induced pluripotent stem (iPS) cells relative to somatic cells (28,29). However, the role of CstF-64 in the cell cycle and in pluripotent stem cells remains unknown.

To study the multiple roles of CstF-64, we examined a mouse embryonic stem cell line in which *Cstf2*, the gene encoding CstF-64, was disrupted by a gene-trap insertion element. Unexpectedly, the *Cstf2* knockout ESCs continued to grow, albeit more slowly and while showing characteristics of differentiation. τCstF-64 expression increased in the *Cstf2* knockout cells. This implied that τCstF-64 partially compensates for CstF-64. High-throughput RNA-sequencing revealed that many replication-dependent histone mRNAs became polyadenylated in the *Cstf2* knockout ESCs cells, suggesting that CstF-64 plays a role in normal 3′ end processing of histone mRNAs. Here we show that CstF-64 is a component of the replication-dependent histone mRNA 3′ end-processing complex in ESCs and that τCstF-64 is recruited to the histone mRNA processing complex only in the absence of CstF-64. Also in its absence, replication-dependent histone mRNAs are polyadenylated to a greater extent. Our results support a model in which CstF-64 controls both the cell cycle and histone mRNA 3′ end processing in stem cells, together resulting in altered pluripotency of these cells.

## MATERIALS AND METHODS

### Cell culture

*Cstf2^Gt(IST10905E6)Tigm^* and *Cstf2^Gt(IST12000G6)Tigm^* cell lines were obtained from Texas A&M Institute for Genomic Medicine (TIGM) and derived from mouse C57BL/6N-derived Lex3.13 ESC lines in which a gene-trap cassette (30) was inserted between the first and second exons (*Cstf2^Gt(IST10905E6)Tigm^*) or the third and fourth exons (*Cstf2^Gt(IST12000G6)Tigm^*). Cells were grown as described (31).

Mouse embryonic stem cells (ESCs) were maintained on 0.1% gelatin-coated 10 cm dishes without feeder cells in Embryo Max Dulbecco's modified Eagle's medium (DMEM) (Millipore) supplemented with 15% ESC-qualified fetal bovine serum (Hyclone/Thermo), 2 mM l-glutamine (Gibco), 0.1 mM -mercaptoethanol (Sigma), 0.1 mM MEM non-essential amino acid stock (Gibco) and 10 ng/mL human leukemia inhibitory factor (LIF, inVitria). ESCs were grown at 37°C in a humidified incubator in 5% CO_2_ and passaged every 2 days (∼70–80% confluency) as described (31). For the generation of the cell proliferation curves, cells were plated in triplicate in 24-well plates at 16 000 cells/well. Cell counts and viability were measured daily using a TC-10 automated cell counter (BioRad). Synchronization of mESCs was adapted from Kapur *et al*. (32). Briefly, mESCs were plated in 6-well dishes for 24 h, followed by treatment with 100 ng/ml Nocodazole (Sigma) for 12 h, followed by release in complete media.

### RNA extraction

RNA was extracted from ESCs using the Qiagen RNAeasy Kit following the manufacturer's instructions. Genomic DNA was eliminated using gDNA spin columns provided in the Qiagen RNAeasy kit. Quality of the RNA was assessed on a 1% agarose gel stained with ethidium bromide (0.5 μg/ml). The quantity of RNA was determined using NanoDrop device.

### Real-time PCR

Complementary DNA was prepared from total mouse ESC RNA by reverse transcription with Super Script II RT (Life Technologies) following the protocol recommended by the manufacturer. For real-time polymerase chain reaction (RT-PCR), 20 ng of cDNA was amplified in triplicates using Power SYBR Green PCR Master Mix (Invitrogen). Polymerase chain reaction program consisted of 95°C for 10 min, 40× cycles of 95°C for 15 s followed by 55°C for 1 min. The ribosomal protein S2 (*Rps2*) served as a loading control and reference gene. Relative expression was calculated using the comparative C_t_ method (33). C_t_ was derived by normalizing the sample mean cycle threshold (C_t_) with its respective control *Rps2* C_t_. The normalized value was subtracted from the control sample to derive the C_t_. C_t_ values were then calculated using the formula 2^−ΔΔ(Ct)^. The presence of gDNA was tested by using –RT controls which had a C_t_ value > 33 cycles. Primers used in this study are listed in Supplemental Table S4.

### Alkaline phosphatase staining

Wild type (WT) and *Cstf2*^E6^ mESCs were washed 2× with phosphate-buffered saline with Tween-20 (PBST), followed by fixation for 5 min at room temperature with fixation reagent (Stemgent). After fixation, cells were stained with alkaline phosphatase (Stemgent) for 15 min at room temperature, then immediately photographed. WT cells cultured without LIF for 96 h were used as negative control.

### Cell cycle analysis

WT and *Cstf2*^E6^ mESCs were prepared for staining by washing with warm Dulbecco's Phosphate-buffered Saline (DPBS) and detached using Accumax (Millipore). Cells were pelleted at 400 × *g* and washed with DPBS supplemented with 0.01% fetal bovine serum (FBS) followed by overnight fixation with 70% ethanol. Following fixation, cells were treated with 40 μg/ml RNase A (Thermo) for 30 min at 37°C and stained with 80 μg/ml propidium iodide (Life Technologies) for 1 h at 4°C. Stained cells were analyzed using a BD LSRII flow cytometer and cell cycle distribution was calculated using FlowJo software.

### Transfection

#### siRNA knockdown

To knockdown CstF-64 and τCstF-64 in WT and *Cstf2*^E6^ cells, 125 pmol of predesigned siRNA (Origene) was transfected using Lipofectamine 2000 (Life Technologies) following the manufacturer's instructions. Knockdown efficiencies were analyzed using western blot. RNA and protein samples were taken after 72 h of transfection.

#### CstF-64 overexpression

To express a 3×FLAG-tagged version of CstF-64, we prepared a custom expression vector on a pcDNA 3.1 backbone, in which we replaced the cytomegalovirus (CMV) promoter with a human elongation factor 1a promoter. The resulting expression plasmid was transfected into mouse embryonic stem and the *Cstf2^E6^* cells using the Xfect reagent (Clontech) following the manufacturer's instructions. Protein and RNA samples were taken 48 h after transfection.

### Western blots

For western blots, protein extracts were prepared by washing and detaching ESCs as previously mentioned. Protein was extracted using RIPA buffer (50 mM Tris-HCl pH:8.8, 150 mM NaCl, 0.1% sodium dodecyl sulphate (SDS), 0.5% deoxycholate and 0.5% NP-40) and the concentrations quantified using the BCA Protein Assay kit (Thermo). Protein were resolved on 10% SDS-polyacrylamide gels and transferred to polyvinylidene difluoride (PVDF) (Millipore) for immunoblot detection. Primary antibodies for all the polyadenylation proteins were purchased from Bethyl Laboratories (Montgomery, TX) with the exception of CstF-64 (3A7) and τCstF-64 (6A9), which were used as described (21). Other antibodies used were rabbit polyclonal anti-FLASH (Millipore), anti-SLBP (Cell Signaling), anti-Histone H2B (Cell Signaling), anti-Cyclin A (Santa Cruz), anti-CDK2 (Cell Signaling) and anti-Histone H3 (Cell Signaling); and mouse monoclonal anti-Cyclin B1 (Millipore), anti-Histone H4 (Cell Signaling), anti-CDK4 (Cell Signaling) and anti-FLAG (Sigma).

### U7 snRNP pull down

U7 snRNP pull down was performed with 10 μg of nuclear extract and streptavidin magnetic beads (Millipore). Briefly, streptavidin beads were washed with 2× B/W buffer (10 mM Tris-HCl (pH 7.5), 1 mM EDTA, 2 M NaCl, 0.1% Tween 20), followed by blocking with 0.20 mg/ml BSA (Thermo) and 0.25 mg/ml yeast tRNA (Thermo). Blocked streptavidin beads were then used to immobilize 1μg RNA oligonucleotide either specific for U7 snRNA (anti-U7) or non-specific (anti-Mock) for 1 h at RT with gentle mixing. Nuclear extracts were then incubated with immobilized streptavidin beads/RNA oligonucleotide in Buffer D and 20 mM EDTA for 1 h at 4°C with gentle mixing. Following incubation, streptavidin magnetic beads were washed three times with Buffer D containing 20 mM EDTA, eluted in sodium dodecylsulphate-polyacrylamide gel electrophoresis (SDS-PAGE) sample buffer at 90°C for 5 min and resolved by SDS-PAGE. The associated proteins with the RNA substrate were identified using western blot. The anti-U7 and anti-mock RNA oligonucleotides were synthesized by Thermo and had the following sequences: Anti-U7 5′-mAmAmAmGmAmGmCmUmGmUmAmAmCmAmCmUmU(18S)(18S)(biotin)-3′; anti-Mock 5′-mCmGmAmGmCmUmCmGmAmUmUmCmGmCmC(18S)(18S)(biotin)-3′. Note that in these sequences, ‘(18S)’ represents an 18-atom spacer and ‘m’ represents the 2′-*O*-methyl group.

### Immunoprecipitation and northern blot

#### Immunoprecipitation

mESCs were lysed on ice for 30 min in RIP buffer (150 mM KCl, 25 mM Tris pH 7.4, 5mM EDTA, 0.5mM DTT, 0.5% NP-40). Protein lysates were then incubated overnight at 4°C with protein A/G magnetic beads (Thermo) coupled to CstF-64 (3A7) monoclonal antibody following manufacturer's instructions. Magnetic beads were washed three times with RIP buffer, followed by SDS-PAGE or RNA extraction with Trizol (Life Technologies) following manufacturer's instructions.

#### Northern blot

2 μg of RNA obtained after IP with 3A7 antibody (21) was run on a 6% urea-poly-acrylamide gel, followed by electrophoretic semi-dry transfer to Nytran nylon membrane (GE). Membrane was UV crosslinked followed by pre-hybridization with ULTRAhyb at 68°C for 1 h. U7 snRNA specific or H3 [α-^32^P]UTP radiolabeled ribo-probes were hybridized overnight at 68°C. The next day the membrane was washed two times for 5 min in 2× SSC and two times for 15 min in 0.1× SSC at 68°C. PCR products corresponding to the U7 snRNA and *Hist1h3c* mRNA were used to produce the radiolabeled ribo-probes using the MAXIscript kit (Life Technologies) with [α-^32^P]UTP.

### RNA-sequencing

Four micrograms of total RNA was used in the preparation of RNA-seq libraries using Illumina's TruSeq RNA Sample Preparation Kits v2 (Illumina Inc.) as recommended. The libraries represent the polyadenylated RNA fraction. RNA-seq libraries were sequenced on HiSeq 2000 platform with 90-nucleotide coverage of each end (PE90). Over 41 million individual sequences from each library were collected with a Q20% larger than 97% (Supplementary Table S5). RNA-seq was performed on two independent biological replicates from wild type mouse ESCs and the *Cstf2^E6^* cells. The reads obtained from each biological replicate were independently aligned on the mouse reference genome (Mouse Genome v37.2, MGSCv37, mm9) using the SeqMan NGen software (DNASTAR Inc., Madison, WI, USA). The assembly files produced by SeqMan NGen were used to determine the differentially expressed genes (2-fold change) using the ArrayStar package (DNASTAR Inc). The *P*-values shown are adjusted *P-*values using Benjamini and Hochberg false discovery rate correction.

### A-seq

A-seq was performed as described (34). Briefly, 40 μg of total RNA was used to isolate the poly(A)^+^ fraction using the Dynabeads mRNA direct Kit (Life Technologies). Poly(A)-selected RNA was partially digested with three different concentration of RNase I (Ambion), re-selected for poly(A) and the 5′ ends were phosphorylated on the Dynabeads. Subsequently, RNAs were eluted and 3′ ends blocked using cordycepin 5′-triphosphate (Sigma) and *Escherichia coli* poly(A) polymerase (New England Biolabs). Simultaneously, the RNAs were treated with RQ1 RNase-Free DNase (Promega). After phenol-chloroform extraction and ethanol precipitation, a 5′ adapter (RA5, Illumina) was ligated. RNAs were reverse transcribed to a complementary DNA using ^32^P labeled primer. cDNAs were resolved on 5% denaturing polyacrylamide gel and the cDNAs between ∼120–150 nt were isolated. PCR was performed with primers adapters similar to the TruSeq Small RNA Sample Preparation Kit (Illumina). Resulting cDNA libraries representative for the formation of the 3′ end of polyadenylated RNAs were sequenced on Illumina platform with 50-nucleotide coverage (SE50). The obtained reads were strand-specific and coincided with the sense strand of the mRNAs. The identical sequences were collapsed into one representative sequence. A-seq libraries were aligned on the mouse reference genome (Mouse Genome v37.2, MGSCv37, mm9) using the SeqMan NGen software (DNASTAR Inc.). The 3′ end cleavage site of polyadenylated histone mRNAs was checked in the assembly file and the first nucleotide (usually an A) that did not align to the genome was recorded as a cleavage site. In the cases that there were As in the genome after the aligned non-A nucleotides, the cleavage site was considered to occur after the A. For generation of Figure 3C, the reads corresponding to the *Hist1h3c* 3′ UTR were aligned and extracted from the A-seq libraries prepared from wild type ESCs and *Cstf2^E6^* cells using local BLAST engine. The extracted sequences (reads) were realigned in the Genome Browser. Subsequently, the corresponding annotated custom tracks were created.

**Figure 1.**
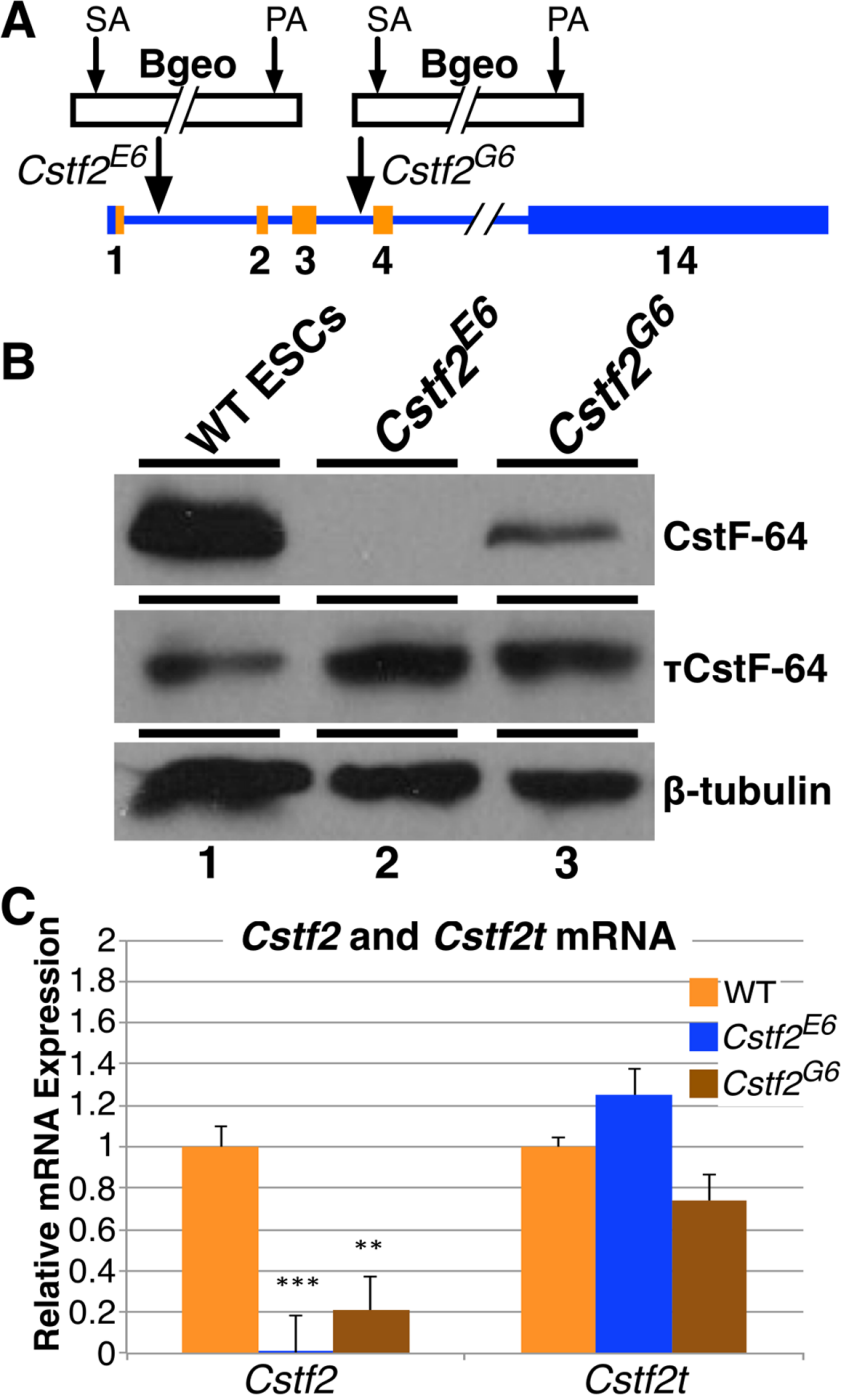
Expression of CstF-64 and τCstF-64 in wild type and *Cstf2* gene-trap interrupted *Cstf2^E6^* and *Cstf2^G6^* mouse embryonic stem cells. (**A**) Schematic representation of insertion of the gene-trap β-galactosidase-neomycin (Bgeo) fusion protein in the first (*Cstf2^E6^*) and third (*Cstf2^G6^*) introns of the *Cstf2* gene in the two respective ESC lines. The gene-trap consists of a splice acceptor (SA) site and polyadenylation (PA) signal. The lighter shade represents the open reading frame of *Cstf2* mRNA. (**B**) Western blot analysis of CstF-64 and τCstF-64 expression in WT (lane 1), *Cstf2*^E6^ (lane 2) and *Cstf2*^G6^ ESCs (lane 3). (**C**) Relative mRNA expression analysis of *Cstf2* and *Cstf2t* mRNAs in the wild type, *Cstf2*^E6^ and *Cstf2*^G6^ ESC lines. ** denotes *P* < 0.01 and *** denotes *P* < 0.001.

**Figure 2.**
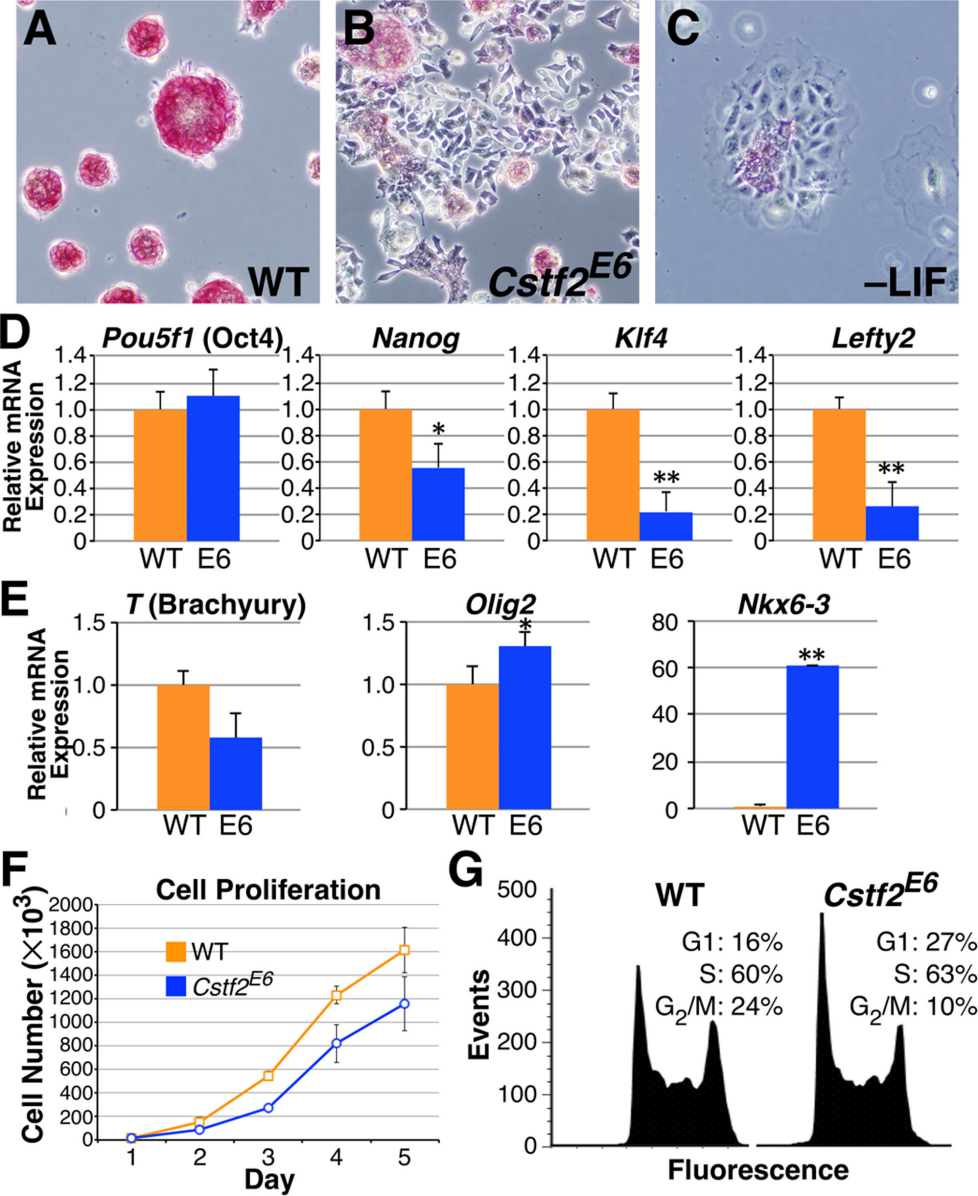
Loss of CstF-64 results in diminished pluripotent state, decreased cell proliferation and disrupted cell cycle. Alkaline phosphatase staining of (**A**) WT ESCs, (**B**) *Cstf2*^E6^ ESCs and (**C**) WT ESCs cultured without LIF for 96 h, 100X magnification. (**D**) Relative mRNA expression of the pluripotency regulators, *Pou5f1* (*Oct4*), *Nanog*, *Klf4* and *Lefty2* and (**E**) differentiation markers, *T* (Brachyury), *Olig2* and *Nkx6-3*, representing mesoderm, ectoderm and endoderm germ layers, respectively. * denotes *P* < 0.05 and ** denotes *P* < 0.01. (**F**) Growth rate analysis of WT and *Cstf2*^E6^ ESCs. Cells were plated in triplicate and data points represent the average cell count. Standard deviation is also shown. The growth rates on days 3, 4 and 5 between the WT and *Cstf2^E6^* ESCs were statistically significant at *P* < 0.05 using a one-tailed *t*-test. (**G**) Cell cycle analysis of WT and *Cstf2*^E6^ ESCs stained with PI and analyzed using flow cytometry. For the cell cycle analysis, a two-tailed *t-*test was performed to obtain significance on triplicate samples. The *P* values for both G_1_ and G_2_/M phases is *P* < 0.05.

**Figure 3.**
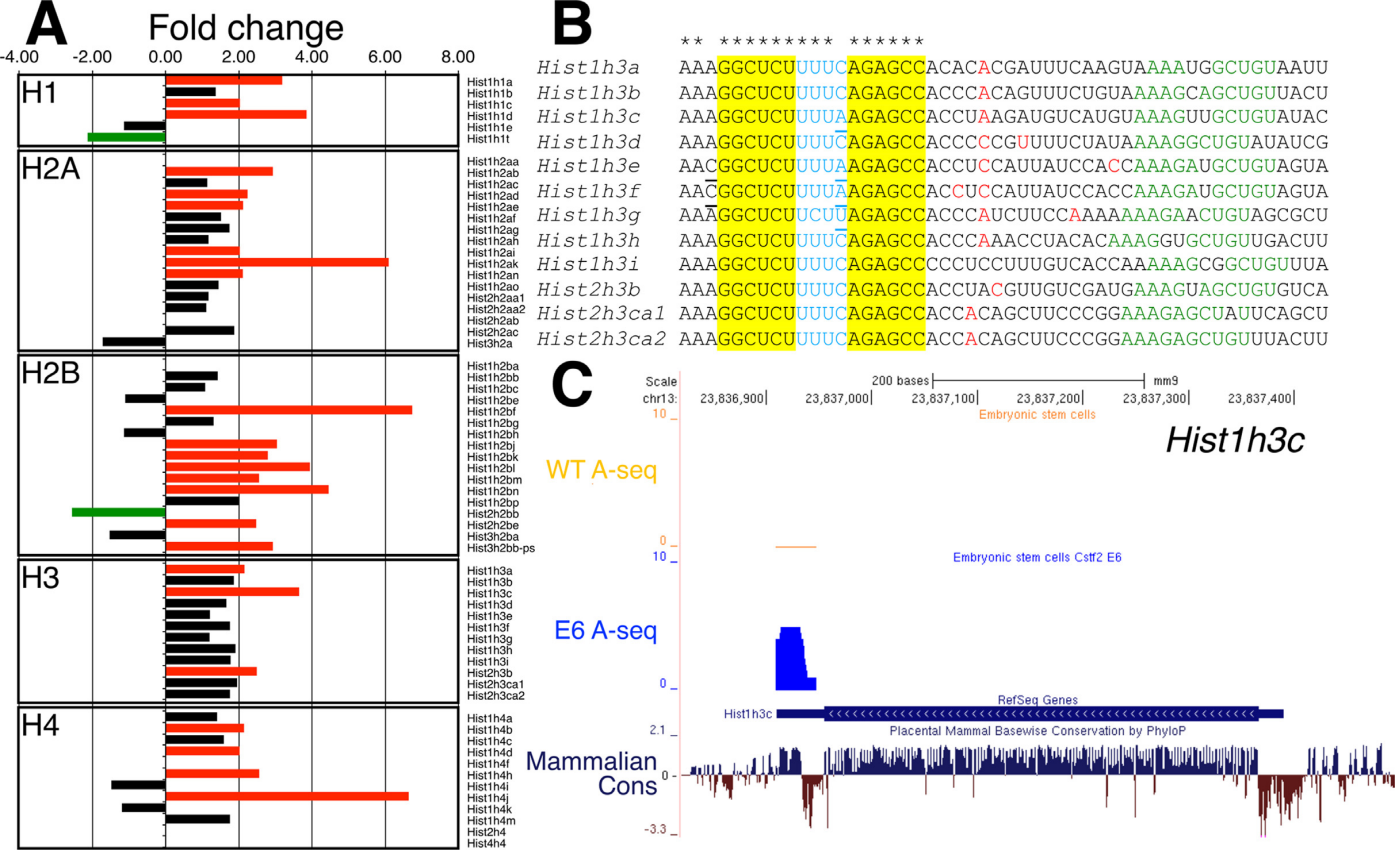
Increase in the polyadenylation of replication-dependent histone mRNAs in *Cstf2^E6^* cells. (**A**) Fold change of the polyadenylated replication-dependent histones families in the *Cstf2^E6^* ESCs versus wild type ESCs. Green bars indicate 2-fold or more down-regulation, red bars—2-fold or more up-regulation and black bars no change (<2-fold) of the expression of the polyadenylated histone mRNAs. (**B**) Cleavage and polyadenylation sites in the replication-dependent histone H3 family. Alignment of the stem-loop region, 3′ end processing cleavage site and downstream genomic sequences. Red nucleotides indicate the 3′ end cleavage and polyadenylation sites as determined by A-seq in wild type ESCs and *Cstf2^E6^* cells. The stem-loop region of the H3 histone mRNAs are highlighted in yellow and blue, respectively. Underlined nucleotides indicate the different nucleotides from the consensus sequence. Asterisks at the top of the alignment point out to the consensus sequence of the stem-loop. Green nucleotides show the respective HDE sequence (AAAGAGCUGU). (**C**) A-seq reads mapped to the mouse genome (mm9) for wild type and *Cstf2^E6^* ESCs. Position of the *Hist1h3c* is shown in the RefSeq Genes track. The blue peak above the RefSeq Genes track is the number of non-normalized reads uniquely mapping to histone *Hist1h3c* obtained from A-seq for the *Cstf2^E6^* cells overlapping with the stem-loop region. Above in orange—A-seq reads obtained from the wild type ESCs. The sequence conservation of *Hist1h3c* in placental mammals is shown at the bottom of the figure.

## RESULTS

### A mouse ESC line that does not express CstF-64

To study the role of CstF-64 in mouse ESCs, we obtained two mouse C57BL/6N-derived Lex3.13 ESC lines in which a gene-trap cassette (30) was inserted between the first and second exons (*Cstf2^Gt(IST10905E6)Tigm^*, herein *Cstf2^E6^*) or the third and fourth exons (*Cstf2^Gt(IST12000G6)Tigm^*, herein *Cstf2^G6^*) of *Cstf2* (Figure 1A). Because the Lex3.13 line is male, only the single X-linked copy of *Cstf2* needed to be inactivated (21). Western blot and quantitative RT-PCR (qRT-PCR) analyses revealed that both *Cstf2^E6^* and *Cstf2^G6^* displayed differential reduction of CstF-64 (Figure 1B, C); CstF-64 protein was undetectable in the *Cstf2^E6^* cell line, whereas the *Cstf2^G6^* cell line displayed only diminished expression (Figure 1B). In addition, qRT-PCR analysis revealed that *Cstf2* mRNA expression was reduced to 0.5% and 21% of wild type levels in the *Cstf2^E6^* and *Cstf2^G6^* cell lines, respectively (Figure 1C). Because they appeared to be amorphic for CstF-64, we decided to perform our further experiments on the *Cstf2^E6^* cells. We received both cell lines at passage 18 and have maintained *Cstf2^E6^* for at least 60 passages without morphological changes.

### τCstF-64 is expressed in wild type ESCs and increases in *Cstf2^E6^* cells

We were surprised that the *Cstf2^E6^* and *Cstf2^G6^* cell lines grew so robustly with absent or reduced expression of CstF-64. Therefore, we asked whether the testis-expressed paralog of CstF-64, τCstF-64 was expressed in these cells. In mammalian spermatogenesis, τCstF-64 compensates for functions of CstF-64 (25), and might perform the same functions in ESCs. We found that τCstF-64 was expressed in wild type mouse ESCs and increased in the *Cstf2^E6^* and *Cstf2^G6^* cells (Figure 1B). We speculate that the increased expression of τCstF-64 is a compensatory mechanism that is activated when CstF-64 expression decreases (24,35,36).

### Loss of CstF-64 in mouse ESCs results in differentiation and decreased expression of markers for pluripotency

Compared to wild type mouse ESCs (Figure 2A), the *Cstf2^E6^* cells displayed a different phenotype that consisted of a flattened morphology and decreased propensity to grow in tightly packed clusters (Figure 2B). To assess whether these morphological differences were accompanied by changes in pluripotency markers, we stained wild type and *Cstf2^E6^* ESCs with alkaline phosphatase, an enzyme that is not expressed in differentiated ESCs (37). Wild type ESCs displayed intense staining for alkaline phosphatase, while the *Cstf2^E6^* cells displayed less staining, suggesting diminished pluripotency (Figure 2A, B). This reduction is similar to wild type ESCs grown in the absence of LIF for 96 h displayed reduced staining (Figure 2C).

To examine additional pluripotency markers, we measured mRNA levels of four stem cell state regulatory genes, *Pou5f1* (Oct4), *Klf4, Nanog* and *Lefty2* (Figure 2D). Relative to wild type ESCs, the *Cstf2^E6^* cells had significantly decreased expression of *Klf4*, *Nanog* and *Lefty2* mRNAs. *Pou5f1* did not change significantly, however, suggesting that not all markers for pluripotency and self-renewal are affected to the same extent in *Cstf2^E6^* cells. In addition, we analyzed the expression of differentiation markers *T* (Brachyury), *Olig2* and *Nkx6-3*, representing the ectoderm, endoderm and mesoderm, respectively. *Cstf2^E6^* cells displayed increased expression of both *Olig2* and *Nkx6-3* mRNAs, indicating increased differentiation to both ectoderm and endoderm lineages (Figure 2E). We observed little or no significant change in *T*, suggesting that loss of CstF-64 may not promote differentiation to the mesoderm lineage.

### *Cstf2^E6^* embryonic stem cells have altered cell cycle and decreased proliferation

ESCs have a short G_1_ phase that is thought to support the pluripotent state through inhibition of pro-differentiation signals. However, the proteins that regulate the cell cycle have not been identified. Previously, it was noted that CstF-64 expression changed as much as 5-fold between G_0_ and S phase in mouse 3T6 fibroblasts (38). Similarly, reduction of CstF-64 to 5% of wild type levels in an avian cell line resulted in G_1_ arrest and apoptosis (39). This suggested that CstF-64 might be involved in cell cycle control. We noticed that *Cstf2^E6^* ESCs grew noticeably slower in culture than wild type ESCs, with an exaggerated initial lag period before reaching exponential growth leading to roughly 35% fewer cells at the end of the 5-day cell proliferation assay (Figure 2F). To test whether the decrease in proliferation was accompanied by changes in the cell cycle, we performed flow cytometry. Wild type mouse ESCs displayed a short G_1_ phase and high proportion of cells in S phase (Figure 2G). In contrast, *Cstf2^E6^* cells displayed a significant increase of cells in G_1_ phase and a decrease of cells in G_2_/M phase (Figure 2G). This suggests that the lack of CstF-64 in the *Cstf2^E6^* cells affects both the G_1_ and G_2_/M phases, points that involve the histone cell cycle control pathway in ESCs (4,6,40–42).

### Loss of CstF-64 results in increased expression of polyadenylated replication-dependent histone mRNAs

To address the source of the cell cycle changes observed, we performed high-throughput RNA-sequencing on wild type and *Cstf2^E6^* ESCs. Puzzlingly, neither the RNA-seq (Supplemental Table S1) nor western blotting (Supplemental Figure S1) data revealed significant changes in the typical G_1_/S and G_2_/M phase regulators such as *Ccnd1, Ccne1, Ccnb1, Ccna1, Ccna2*, *Cdk2*, *Cdk4* and *Cdk6*. Gene ontology (GO) determined that the most significant enriched processes for up-regulated genes included nucleosome assembly, chromatin assembly and protein-DNA complex assembly (Table 1). We also found an increase in the polyadenylated forms of many of the replication-dependent histone mRNAs in the *Cstf2^E6^* cells, including genes from the H2A, H2B, H3, H4 and H1 families (Figure 3A and Supplemental Table S2).

**Table 1.** GO terms derived from RNA-seq data in WT and *Cstf2^E6^* ESCs

GO ID	GO name	*P*-value
Enriched for downregulated genes		
GO: 0006811	Ion transport	1.10E−08
GO: 0006955	Immune response	1.90E−08
GO: 0007155	Cell adhesion	2.60E−07
GO: 0022610	Biological adhesion	2.80E−07
GO: 0030001	Metal ion transport	3.80E−07
Enriched for upregulated genes		
GO: 0006334	Nucleosome assembly	1.60E−09
GO: 0031497	Chromatin assembly	2.40E−09
GO: 0034728	Nucleosome organization	2.90E−09
GO: 0065004	Protein-DNA complex assembly	2.90E−09
GO: 0006333	Chromatin assembly or disassembly	1.10E−08
GO: 0006323	DNA packaging	2.40E−08

Similarly, we mapped the cleavage and polyadenylation (C/P) sites and their frequency in the wild type and *Cstf2^E6^* ESCs by high-throughput 3′ end poly(A) sequencing (A-seq, (34)). A-seq for the replication-dependent histones did not reveal changes in the locations of C/P sites in the *Cstf2^E6^* cells compared to wild type ESCs (Figure 3B and Supplemental Table S3). However, in agreement with the RNA-seq data, we observed an increase in A-seq reads for some of the replication-dependent histone mRNAs in the *Cstf2^E6^* cells (Figure 3C, Supplemental Table S3).

**Figure 4.**
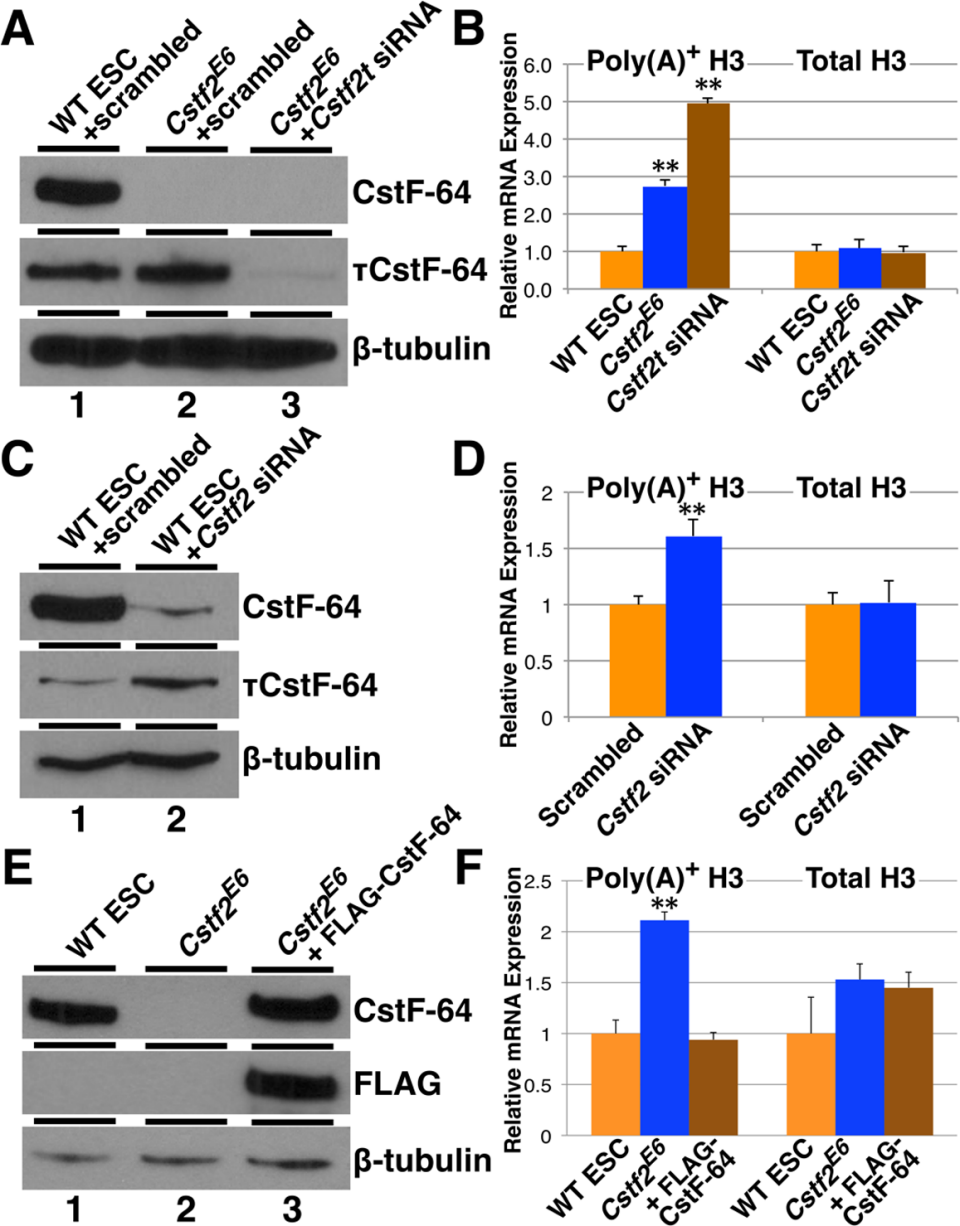
CstF-64 and τCstF-64 are necessary for normal 3′ end processing of replication-dependent histone mRNAs. (**A**) Western blot analysis of CstF-64 and τCstF-64 expression in WT (lane 1), *Cstf2*^E6^ ESCs transfected with scrambled siRNA (lane 2) and *Cstf2*^E6^ ESCs transfected with siRNA specific for *Cstf2t* gene (lane 3). (**B**) Relative mRNA expression analysis of polyadenylated and total *Hist1h3c* histone mRNA in wild type ESCs (orange), *Cstf2*^E6^ cells transfected with scrambled siRNA (blue) or *Cstf2*^E6^ cells transfected with siRNA against *Cstf2t* gene (brown). (**C**) Western blot analysis of CstF-64 and τCstF-64 expression in WT ESCs either transfected with scrambled siRNA (lane 1) or *Cstf2* gene specific siRNA (lane 2). (**D**) Relative mRNA expression analysis of polyadenylated and total *Hist1h3c* histone mRNA in wild type ESCs transfected with scrambled (orange) or *Cstf2* gene specific siRNA (blue). (**E**) Western blot analysis of CstF-64 expression in WT ESCs (lane 1), *Cstf2*^E6^ ESCs (lane 2) and *Cstf2*^E6^ ESCs ectopically expressing CstF-64 (lane 3). (**F**) Corresponding relative mRNA expression analysis of polyadenylated and total *Hist1h3c* histone mRNA. * denotes *P* < 0.05 and ** *P* < 0.01.

To assess polyadenylated histone mRNA levels directly, we used quantitative RT-PCR (qRT-PCR) to compare total histone mRNA levels (random oligo-primed) and polyadenylated histone mRNA levels (oligo(dT)-primed) for *Hist1h3c* (H3). We observed increased expression of polyadenylated H3 in the *Cstf2^E6^* cells compared to wild type cells, but no change in total H3 mRNA levels (Figure 4B). To determine whether τCstF-64 played a role in this process, we knocked down *Cstf2t*, the gene that encodes τCstF-64 with a specific siRNA (Figure 4A, B). Depletion of τCstF-64 in the *Cstf2^E6^* cells resulted in an even greater increase in the amount of polyadenylated H3 mRNA (Figure 4B), whereas siRNA knockdown of τCstF-64 in wild type cells did not result in a change (Supplemental Figure S2). This suggested that the effect of τCstF-64 on histone mRNA processing did not become evident except when CstF-64 was reduced or missing. To test whether the observed increase in polyadenylated histone mRNAs was an artifact of the gene-trap insertion, we knocked down CstF-64 in wild type cells using siRNA specific for *Cstf2* (Figure 4C). Consistent with the *Cstf2^E6^* ESCs, we observed a significant increase in polyadenylated H3 mRNA (Figure 4D). Finally, we expressed CstF-64 transiently in the *Cstf2*^E6^ cells (Figure 4E). Within 48 h of CstF-64 expression, we were able to completely reduce the abnormal polyadenylation of replication-dependent histone genes in the *Cstf2*^E6^ cells (Figure 4F). These results suggest a direct role for CstF-64 and, in its absence, τCstF-64 in replication-dependent histone mRNA 3′ end processing.

### CstF-64 is cell cycle regulated in wild type mouse ESCs and τCstF-64 is cell cycle regulated in both wild type and *Cstf2^E6^* cells

**Figure 5.**
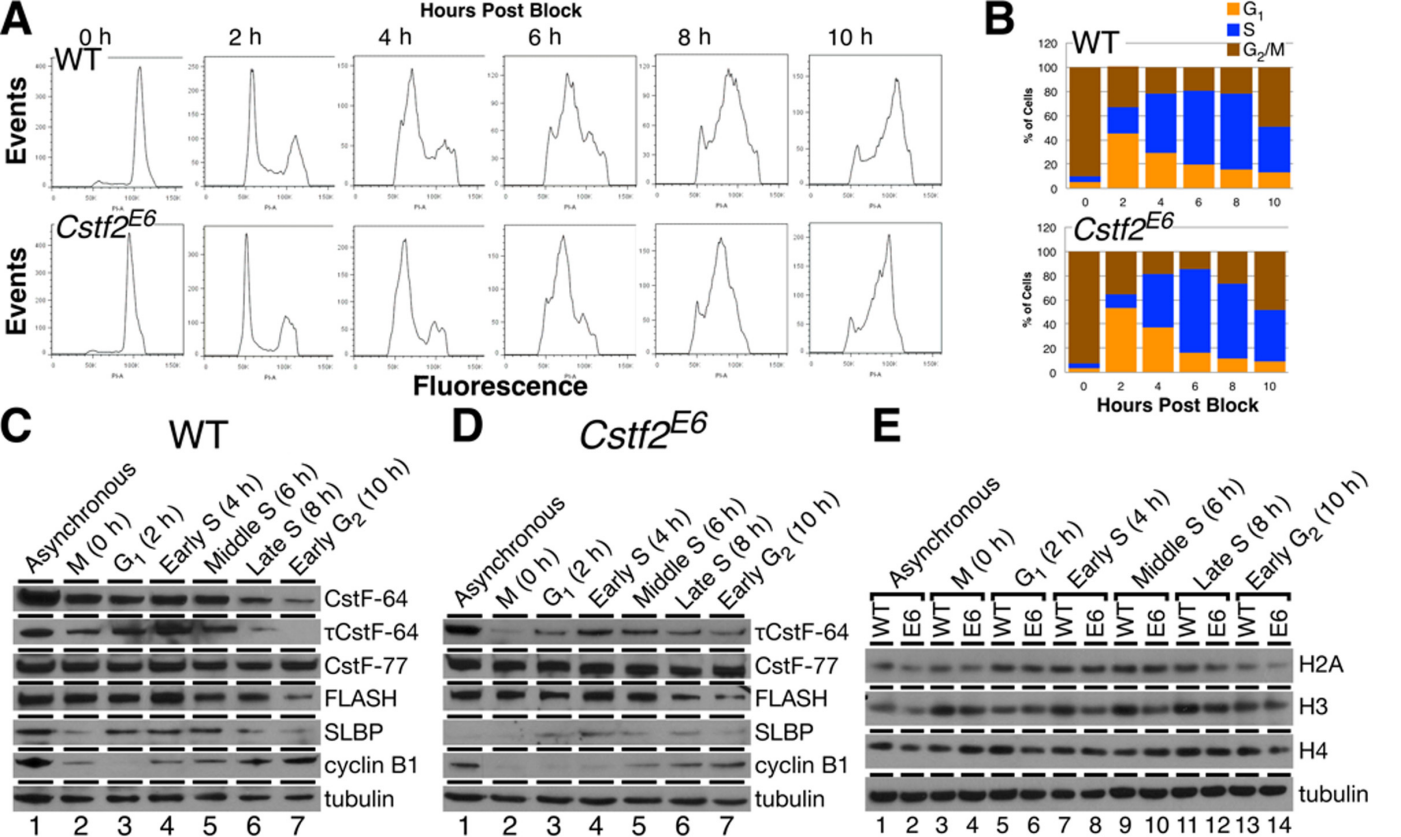
CstF-64 is required for S-phase entry and histone expression. (**A**) Cell cycle analysis of WT and *Cstf2*^E6^ ESCs mitotically synchronized (0 h) with nocodazole and subsequently released for 10 h in 2 h time points (2–10 h). Cells were stained with PI and analyzed using flow cytometry. (**B**) Corresponding percentages of synchronized and released WT and *Cstf2*^E6^ ESCs in the cell cycle phases, G_1_ (orange), S (blue) and G_2_/M (brown). (**C**, **D**) Western blot analysis detecting the expression of CstF-64, τCstF-64, CstF-77 and the histone processing components, FLASH and SLBP in mitotically synchronized WT and *Cstf2*^E6^ ESCs that were released for 10 h post block. Cyclin B1 expression indicates G_2_/M phase transition. (**E**) Comparative western blot analysis of the core histone families, H2B, H3 and H4 in mitotically synchronized WT and *Cstf2*^E6^ ESCs.

Loss of CstF-64 in mouse ESCs seemed to disrupt both proliferation and cell cycle, so we examined cell cycle progression following synchronization of wild type and *Cstf2^E6^* ESCs. After cells were released from nocodazole block, time points were taken every 2 h for 10 h; over 90% of wild type and *Cstf2^E6^* ESCs (Figure 5A, B, 0 h) were synchronized in G_2_/M through G_1_ and into S phase. Compared to wild type cells (Figure 5A and B, 2–6 h), the *Cstf2^E6^* cells displayed a delayed progression into S phase resulting in an increase of cells in G_1_ (Figure 5A and B, 2–6 h), suggesting that cell cycle changes in the *Cstf2^E6^* cells were due to a block or delay in entry into S phase.

Next, we examined expression of proteins associated with polyadenylation and histone mRNA 3′ end processing in these synchronized cells. As a cell cycle marker, cyclin B1 levels were lowest at 2 h after release from nocodazole block and increased until 10 h post-block in both wild type ESCs (Figure 5C) and in *Cstf2^E6^* cells (Figure 5D). However, cyclin B1 expression in the *Cstf2^E6^* cells seemed to be delayed compared to wild type cells, consistent with an increased cell cycle time (Figure 5C and D).

CstF-64 varied throughout the cell cycle in ESCs, with lowest expression in G_2_ and peak expression in early S phase (Figure 5C), in agreement with earlier reports (38). τCstF-64 expression was similar to CstF-64 in wild type ESCs (Figure 5C); that pattern did not change in *Cstf2^E6^* cells (Figure 5D) despite the overall increased τCstF-64 expression in these cells (Figure 1B). This suggests that τCstF-64 is coupled to the cell cycle in the same manner as CstF-64 in both wild type and *Cstf2^E6^* cells. CstF-77 expression was relatively unchanged throughout the cell cycle in both wild type (Figure 5C) and *Cstf2^E6^* cells (Figure 5D), suggesting that both CstF-64 and τCstF-64 are cell cycle-regulated in wild type mouse ESCs, but that other polyadenylation factors are not.

Histone mRNA 3′ end processing factors SLBP and FLASH are cell cycle regulated in ESCs, with peak expression in early S phase (Figure 5C). These patterns do not change in *Cstf2^E6^* cells (Figure 5D). However, while the expression of the H2A and H4 core histones throughout the cell cycle was similar between wild type and *Cstf2^E6^* ESCs, the H3 histone family displayed differential expression (Figure 5E). Histone H3 expression was consistently lower throughout the cell cycle in *Cstf2^E6^* cells than in wild type ESCs (Figure 5E).

### CstF-64 is a component of the U7 snRNP complex in embryonic stem cells

**Figure 6.**
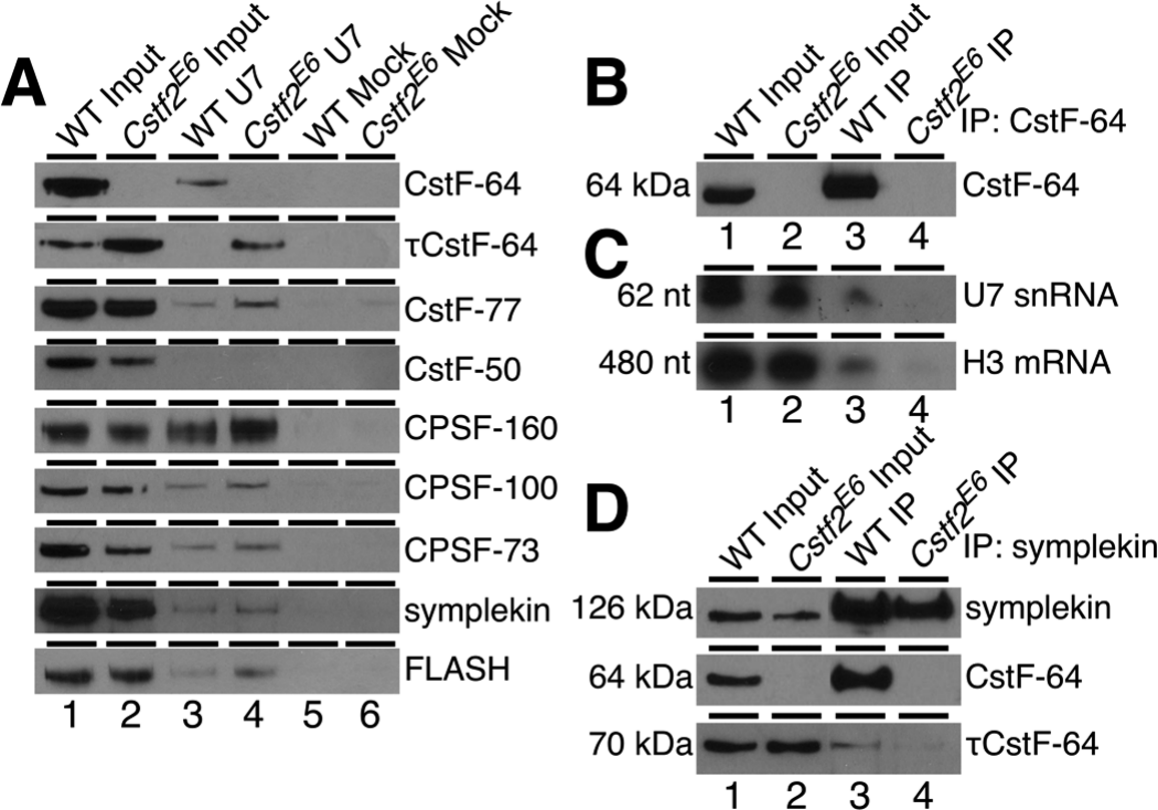
CstF-64 is a component of the replication-dependent histone mRNA 3′ end-processing complex. (**A**) Western blot analysis of the proteins isolated from a pull-down experiment using anti-U7 snRNP oligonucleotide (lanes 3 and 4) or unrelated mock oligonucleotide (lanes 5 and 6) in WT and *Cstf2^E6^* ESCs nuclear extracts. 1/100^th^ of the nuclear extracts from the wild type ESCs (lane 1) and *Cstf2^E6^* cells (lane 2) before the pull down were also loaded on the gel serving as an input control. Antibodies against the indicated proteins were used. (**B**) Western blot analysis of immunoprecipitation using an anti-CstF-64 antibody in wild type ECCs (lane 3) and *Cstf2*^E6^ (lane 4) cells. 1/100th of the total proteins was also loaded as an input control. Wild type ESCs (lane 1) and *Cstf2^E6^* cells (lane 2). (**C**) Northern blot of RNA from immunoprecipitation with antibodies against CstF-64 that were hybridized with radiolabeled ribo-probes specific for U7 snRNA or *Hist1h3c* mRNA. Lane 1, 2 μg of total RNA from wild type ESCs; lane 2, 2 μg of total RNA from *Cstf2^E6^* cells; lanes 3–4, 2 μg of RNA purified from CstF-64 immunoprecipitation from wild type ESCs (lane 3) or *Cstf2^E6^* cells (lane 4). (**D**) Western blot analysis of immunoprecipitation with an anti-symplekin antibody in wild type (lane 3) and *Cstf2^E6^* (lane 4) ESCs protein lysates. IP precipitates from wild type and *Cstf2^E6^* ESCs were probed for interaction with CstF-64 and τCstF-64 as indicated.

The data presented above support the involvement of CstF-64 in replication-dependent histone mRNA 3′ end processing. To address the mechanism of this involvement, we tested whether CstF-64 was a component of the histone mRNA 3′ end-processing machinery. We isolated U7 snRNP complexes from wild type or *Cstf2^E6^* ESC nuclear extracts using a U7-complementary oligonucleotide (anti-U7) according to Yang *et al*. (15). Using this method, we found that FLASH bound to the anti-U7 oligonucleotide but not to the anti-mock oligonucleotide in extracts from wild type ESCs (Figure 6A, lanes 3 and 5). This demonstrated enrichment of U7-containing histone mRNA 3′ end processing complexes. Similarly, we found FLASH in *Cstf2^E6^* cell extracts with the anti-U7 oligo but not with the anti-mock oligo, suggesting that the histone mRNA 3′ end-processing complex forms in cells lacking CstF-64 (Figure 6, lanes 4 and 6). Symplekin, CPSF-160, CPSF-100, CPSF-73 and CstF-77 were also associated with the U7 snRNA in wild type ESC and *Cstf2^E6^* cell extracts, whereas CstF-50 did not seem to be associated (Figure 6A, lanes 3 and 4).

### τCstF-64 is recruited to the U7 snRNP complex in the absence of CstF-64

We did not detect τCstF-64 associated with U7 in extracts from wild type ESCs (Figure 6A, lane 3). However, we observed considerable recruitment of τCstF-64 to the histone mRNA 3′ end-processing complex in the *Cstf2^E6^* cell extracts (Figure 6A, lane 4). This suggests that τCstF-64 can participate in some of the non-polyadenylation functions of CstF-64 in the absence of the latter. More specifically, CstF-64 is recruited to the U7 snRNP complex in mouse ESCs along with other proteins involved in the polyadenylation process, but τCstF-64 can be recruited to the complex only when CstF-64 is not present.

### Components of the U7 snRNP complex and histone mRNAs are associated with CstF-64 in embryonic stem cells

To further confirm that CstF-64 associates with the histone mRNA 3′ end-processing complex, we performed immunoprecipitation from wild type ESC and *Cstf2^E6^* cell extracts using an anti-CstF-64 antibody (Figure 6B), and then probed the precipitate with a radiolabeled probe for the U7 snRNA (Figure 6C). Immunoprecipitation with anti-CstF-64 enriched CstF-64 from wild type ESC extract (Figure 6B, lane 3) but not from the *Cstf2^E6^* cell extract (lane 4), as expected. Correspondingly, the U7 snRNA was found in immunoprecipitates from the wild type ESC extract (Figure 6C, lane 3, top panel) but not in the *Cstf2^E6^* extract (lane 4, top panel). To test whether the interaction of CstF-64 and U7 snRNA was functional, we probed for histone H3 mRNA. Histone H3 mRNA was found in immunoprecipitates from the wild type ESC extract (Figure 6C, lane 3, bottom panel) but not from the *Cstf2^E6^* extract (lane 4, bottom panel). These data demonstrate that CstF-64 associates with the U7 snRNP within the replication-dependent histone mRNA 3′ end-processing complex.

It has been shown that both CstF-64 and symplekin are necessary to link FLASH and Lsm11 with the other polyadenylation factors in the histone mRNA 3′ end processing complex (15,18). Since τCstF-64 is recruited to the complex in the absence of CstF-64, we tested whether it interacted with symplekin by co-immunoprecipitation in the wild type and *Cstf2^E6^* cells. Using an anti-symplekin antibody, we enriched for symplekin in both wild type ESCs and *Cstf2^E6^* cells (Figure 6D). CstF-64 was also enriched in the IP fraction in wild type cells, suggesting a robust interaction between the two proteins. In contrast, we did not observe the same strong interaction of symplekin with τCstF-64 in wild type and *Cstf2^E6^* cells (Figure 6D, lanes 3, 4), suggesting that τCstF-64 does not interact with symplekin like CstF-64, and further suggests a different recruitment mechanism for τCstF-64 to the U7 snRNP complex.

## DISCUSSION

The therapeutic use of embryonic stem cells requires a fundamental understanding of features of the stem cells that promote pluripotency, self-renewal and differentiation. The lack of R point in the ESC cell cycle appears to be a distinctive feature of these cells, and suggests reliance on other G_1_/S phase regulation mechanisms. The abbreviated cell cycle observed in ESCs is thought to maintain stem cell identity through a shortened G_1_ phase that acts as a blockade for pro-differentiation signals (1–5). Uniquely, ESCs traverse the G_1_/S phase transition by regulating expression of replication-dependent histones (4,6,41), and specific histone genes are associated with pluripotency (8). Here, we show that CstF-64 is required for the maintenance of pluripotency and cell cycle progression in mouse ESCs. In the absence of CstF-64, ESCs grow more slowly, undergo partial differentiation and display an altered cell cycle. As a potential driver for these changes, we observed an increase in the expression of processed histone mRNAs and a reduction in expression of key histones, resulting in a delay in entering S phase. We further showed that CstF-64 is a component of the histone mRNA 3′ end-processing complex in ESCs. In the absence of CstF-64 in these cells, the paralogous protein τCstF-64 is incorporated into the complex, and polyadenylation of histone mRNAs is observed. Therefore, an important role of CstF-64 in ESCs appears to be *suppression* of polyadenylation in histone mRNA 3′ end processing while favoring U7-directed cleavage. This role of CstF-64 may also regulate the cell cycle at the G_1_/S phase transition, although that mechanism is not yet understood.

Initially, we were quite surprised that the *Cstf2^E6^* cells could continue to grow without detectable CstF-64. Studies in non-mammalian species showed that *Cstf2* was essential for viability and growth (39,43,44), and we expected the same in ESCs. However, ESCs also express τCstF-64 (Figures 1 and 3, and (24)), which suggests that τCstF-64 can take on some of the functions of CstF-64 in the *Cstf2^E6^* cells. However, τCstF-64 does not perfectly complement CstF-64 function, since the *Cstf2^E6^* cell phenotype differs from that of wild type ESCs. The primary function of τCstF-64 is to support spermiogenesis (25,26,45). These new results suggest that, while τCstF-64 can support some of the functions of CstF-64, it is not optimal to support all those functions. This further suggests that there are germ cell functions of τCstF-64 still to be discovered.

We were also surprised that loss of CstF-64 resulted in increased polyadenylation of replication-dependent histone mRNAs, decreased pluripotency and changes in the ESC cell cycle due to defects in the transition into S phase. Studies in non-pluripotent cells have demonstrated multiple roles for CstF-64 in mRNA processing, including roles involving 3′ end definition and intron binding (24,36). Similar wide-ranging functions have been associated with τCstF-64 in germ cells (46). Roles for CstF-64 in histone mRNA processing have been demonstrated, including participation of CstF-64 in the histone mRNA 3′ end processing complex (15,17–19) and changes in histone mRNA abundance in cells having reduced CstF-64 (35). Ironically, our results suggest that at least one role of CstF-64 is to prevent polyadenylation of histone mRNAs while promoting stem-loop 3′ end processing.

Previous studies demonstrated that depletion of proteins involved in histone mRNA 3′ end processing and transcription, including cyclin-dependent kinase 9 (CDK9), RING finger protein 20 (RNF20), RNF40, NPAT/p220, negative elongation factor-E (NELF-E), U7 snRNA and SLBP resulted in an increase in the level of polyadenylated histone transcripts (10,47–50). Furthermore, inhibition of DNA replication stimulated the production of polyadenylated histones as well as a G_1_ arrest (10,12). Similarly, reduction of the core replication-dependent histone mRNA processing components, Lsm10, Lsm11 and Zfp100 result in an increase of cells in G_1_ phase due to a partially inactive U7 snRNP (40). This implies that the role of CstF-64 in histone mRNA 3′ end processing may involve specific interactions with symplekin, FLASH and Lsm11 within the U7 snRNP complex (15,17,18,35). We hypothesize that the decreased expression of histone proteins is the result of increased polyadenylation of histone mRNAs in the *Cstf2^E6^* cells (Figure 3A), and thus explains the cell cycle changes observed there. The apparent lack of change in H2A and H4 histone levels may be due to the broad specificity of the antibodies used.

**Figure 7.**
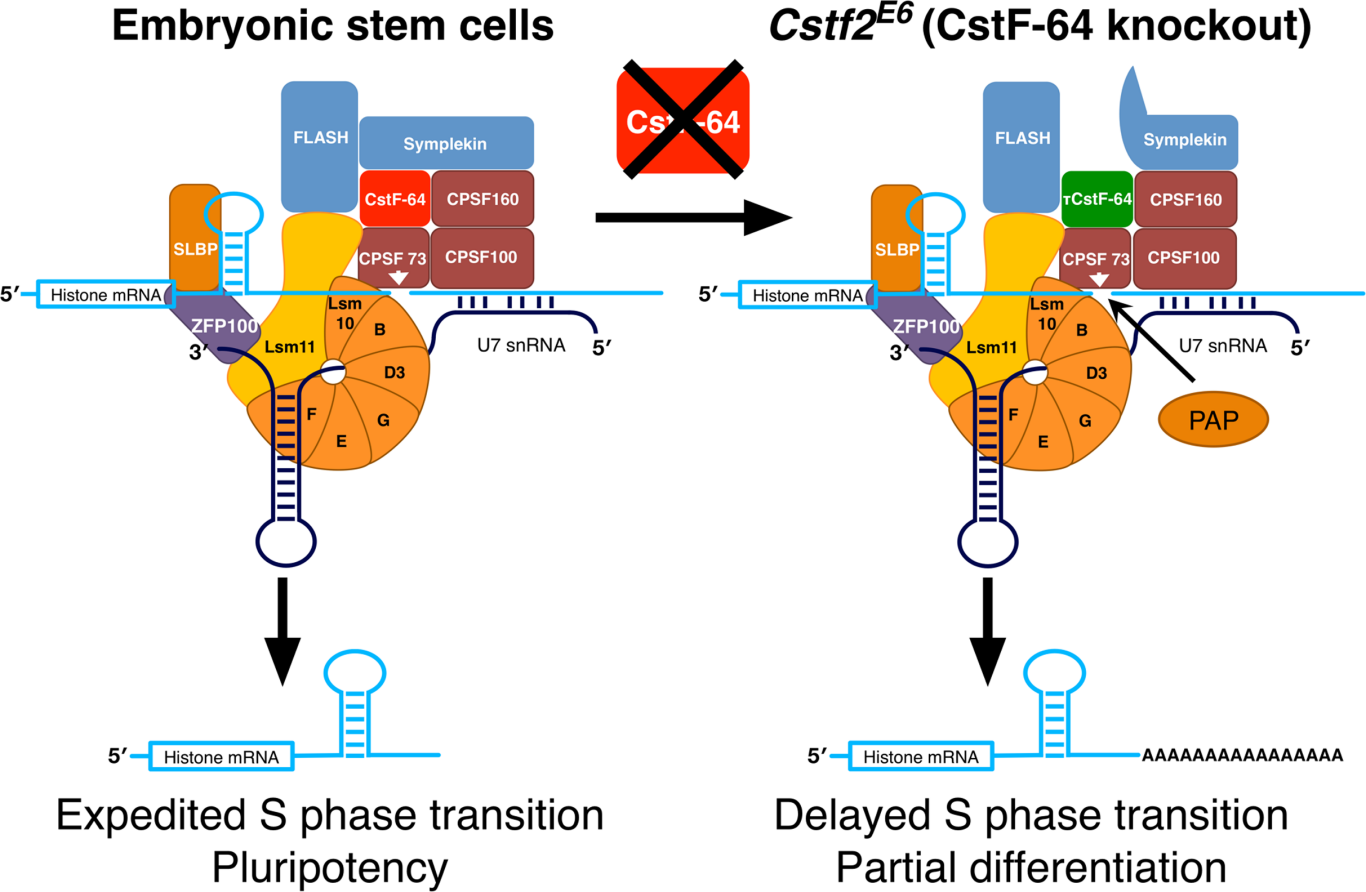
Schematic representation of how depletion of CstF-64 increases polyadenylation of replication-dependent histone mRNAs and modulates the cell cycle in ESCs and therefore pluripotency. The panel on the left describes the histone mRNA 3′ end processing complex in wild type ESCs. On the right: modified histone mRNA 3′ end processing complex in the *Cstf2^E6^* (CstF-64 knockout) cells. Histone mRNA cleavage occurs in wild type mouse ESCs due to interactions of the U7 snRNP with the histone mRNA downstream element (left panel). Other proteins involved in mRNA polyadenylation further associate with the complex, including CstF-64 and symplekin. The complex promotes site-specific cleavage of the histone mRNA by CPSF-73. Together, these processes correlate with normal entry into S-phase and pluripotency. In normal ESCs, a small amount of these cleaved transcripts are polyadenylated. In *Cstf2^E6^* cells (right panel), CstF-64 is absent, resulting in recruitment of τCstF-64 to the histone 3′ end processing complex (although τCstF-64 interacts more poorly with symplekin). This results in an increase in polyadenylation of the cleaved histone transcripts, presumably by poly(A) polymerase (PAP).

We found that CstF-64 interacts more strongly with symplekin than does τCstF-64 (Figure 6D) (24). Others have shown that the CstF-64/symplekin interaction is essential to link FLASH and Lsm11 with the other CPSF factors in the histone mRNA 3′ end-processing complex (15,18). Based on these data, we propose a model in which interaction of CstF-64 with symplekin inhibits the polyadenylation of replication-dependent histone mRNAs in wild type ESCs, supporting both pluripotency and progression through the histone cell cycle checkpoint into S phase while suppressing differentiation. Our data also demonstrate a stronger recruitment of certain CPSF and CstF factors to the replication-dependent histone mRNA 3′ end processing complex in the absence of CstF-64, including CPSF-100, CPSF-160 and CstF-77 (Figure 6A). The increased recruitment of these factors in the *Cstf2^E6^* cells could result in a U7 snRNP complex that favors polyadenylation of histone mRNAs instead of the usual histone stem loop processing (Figure 7).

Further reduction of τCstF-64 in the *Cstf2^E6^* cells results in a greater degree of polyadenylated histone mRNAs (Figure 4A). This suggests that the default processing of histone mRNAs is polyadenylation, and that entry into the histone stem-loop processing pathway requires CstF-64. A similar alternative pathway to polyadenylation appears to be present in Drosophila replication-dependent histone mRNAs (51–53). Mammalian testis histone variants are also polyadenylated (54,55) as are histone mRNAs in yeast (56), demonstrating that this mechanism is ancient and conserved (57,58).

LIF withdrawal results in a lengthening of G_1_ phase which occurs prior to the appearance of lineage-specific markers (2). In addition, cell-fate decisions are tightly associated with cell cycle machinery and length of G_1_ phase (1). Our data demonstrate a role for CstF-64 regulating the length of the G_1_ phase, consequently allowing the induction of differentiation and decreased pluripotency of the *Cstf2*^E6^ cells possibly through the histone-dependent cell cycle checkpoint in mouse ESCs. We speculate that this checkpoint may coexist with the cyclin-based mechanisms in non-ESCs. For example, reduction of CstF-64 in an avian cell line blocked those cells from entering S phase (39), supporting the role of CstF-64 in G_1_/S checkpoint control. Our own unpublished experiments show similar results in several somatic cell lines (not shown). However, an opposite effect—increase in S and G_2_/M phases at the expense of G_1_—was noted when CstF-64 and τCstF-64 were knocked down in HeLa cells (35). This suggests that CstF-64 may be involved in both ESC and somatic cell cycle control, probably through distinct mechanisms. Reduced levels of CstF-64 in G_2_/M (Figure 4) may further hint at a role for that protein in G_2_ or metaphase checkpoints (38).

Finally, loss of *Cstf2* results in partial, though incomplete and undirected differentiation of ESCs. While this phenotype can be attributed to changes in histone dynamics resulting in changes to the hyperactive chromatin state or cell cycle, it is also likely that CstF-64 is required for expression of genes involved in specific differentiation pathways. This conclusion is supported by the reduction in pluripotency markers and the increase in ectodermal and endodermal, though not mesodermal markers (Figure 2). In other experiments, we have been able to differentiate *Cstf2^E6^* cells toward ectodermal and mesodermal, but not endodermal endpoints (B. A. Y., C. C. M., manuscript in preparation), suggesting that the different developmental lineages have markedly different requirements for CstF-64.

## SUPPLEMENTARY DATA

Supplementary Data are available at NAR Online.

SUPPLEMENTARY DATA

## References

[1] Pauklin S., Vallier L. (2013). The cell-cycle state of stem cells determines cell fate propensity. Cell.

[2] Coronado D., Godet M., Bourillot P.Y., Tapponnier Y., Bernat A., Petit M., Afanassieff M., Markossian S., Malashicheva A., Iacone R. (2013). A short G1 phase is an intrinsic determinant of naive embryonic stem cell pluripotency. Stem Cell Res..

[3] Ghule P.N., Medina R., Lengner C.J., Mandeville M., Qiao M., Dominski Z., Lian J.B., Stein J.L., van Wijnen A.J., Stein G.S. (2011). Reprogramming the pluripotent cell cycle: restoration of an abbreviated G1 phase in human induced pluripotent stem (iPS) cells. J. Cell. Physiol..

[4] Becker K.A., Stein J.L., Lian J.B., van Wijnen A.J., Stein G.S. (2007). Establishment of histone gene regulation and cell cycle checkpoint control in human embryonic stem cells. J. Cell. Physiol..

[5] Koledova Z., Kafkova L.R., Calabkova L., Krystof V., Dolezel P., Divoky V. (2010). Cdk2 inhibition prolongs G1 phase progression in mouse embryonic stem cells. Stem Cells Dev..

[6] Medina R., Ghule P.N., Cruzat F., Barutcu A.R., Montecino M., Stein J.L., van Wijnen A.J., Stein G.S. (2012). Epigenetic control of cell cycle-dependent histone gene expression is a principal component of the abbreviated pluripotent cell cycle. Mol. Cell. Biol..

[7] Ghule P.N., Dominski Z., Yang X.C., Marzluff W.F., Becker K.A., Harper J.W., Lian J.B., Stein J.L., van Wijnen A.J., Stein G.S. (2008). Staged assembly of histone gene expression machinery at subnuclear foci in the abbreviated cell cycle of human embryonic stem cells. Proc. Natl. Acad. Sci. U.S.A..

[8] Yang L., Duff M.O., Graveley B.R., Carmichael G.G., Chen L.L. (2011). Genomewide characterization of non-polyadenylated RNAs. Genome Biol..

[9] Zhang Y., Cooke M., Panjwani S., Cao K., Krauth B., Ho P.Y., Medrzycki M., Berhe D.T., Pan C., McDevitt T.C. (2012). Histone h1 depletion impairs embryonic stem cell differentiation. PLoS Genet..

[10] Pirngruber J., Johnsen S.A. (2010). Induced G1 cell-cycle arrest controls replication-dependent histone mRNA 3’ end processing through p21, NPAT and CDK9. Oncogene.

[11] Marzluff W.F., Wagner E.J., Duronio R.J. (2008). Metabolism and regulation of canonical histone mRNAs: life without a poly(A) tail. Nat. Rev. Genet..

[12] Kari V., Karpiuk O., Tieg B., Kriegs M., Dikomey E., Krebber H., Begus-Nahrmann Y., Johnsen S.A. (2013). A Subset of histone H2B genes produces polyadenylated mRNAs under a variety of cellular conditions. PLoS One.

[13] Shepard P.J., Choi E.A., Lu J., Flanagan L.A., Hertel K.J., Shi Y. (2011). Complex and dynamic landscape of RNA polyadenylation revealed by PAS-Seq. RNA.

[14] Yang X.C., Torres M.P., Marzluff W.F., Dominski Z. (2009). Three proteins of the U7-specific Sm ring function as the molecular ruler to determine the site of 3’-end processing in mammalian histone pre-mRNA. Mol. Cell. Biol..

[15] Yang X.C., Sabath I., Debski J., Kaus-Drobek M., Dadlez M., Marzluff W.F., Dominski Z. (2013). A complex containing the CPSF73 endonuclease and other polyadenylation factors associates with U7 snRNP and is recruited to histone pre-mRNA for 3’-end processing. Mol. Cell. Biol..

[16] Ruepp M.D., Vivarelli S., Pillai R.S., Kleinschmidt N., Azzouz T.N., Barabino S.M., Schumperli D. (2010). The 68 kDa subunit of mammalian cleavage factor I interacts with the U7 small nuclear ribonucleoprotein and participates in 3’-end processing of animal histone mRNAs. Nucleic Acids Res..

[17] Sullivan K.D., Steiniger M., Marzluff W.F. (2009). A core complex of CPSF73, CPSF100, and Symplekin may form two different cleavage factors for processing of poly(A) and histone mRNAs. Mol. Cell.

[18] Sabath I., Skrajna A., Yang X.C., Dadlez M., Marzluff W.F., Dominski Z. (2013). 3′-End processing of histone pre-mRNAs in Drosophila: U7 snRNP is associated with FLASH and polyadenylation factors. RNA.

[19] Kolev N.G., Steitz J.A. (2005). Symplekin and multiple other polyadenylation factors participate in 3′-end maturation of histone mRNAs. Genes Dev..

[20] Darmon S.K., Lutz C.S. (2012). mRNA 3′ end processing factors: a phylogenetic comparison. Comp. Funct. Genomics.

[21] Wallace A.M., Dass B., Ravnik S.E., Tonk V., Jenkins N.A., Gilbert D.J., Copeland N.G., MacDonald C.C. (1999). Two distinct forms of the 64,000 M*_r_* protein of the cleavage stimulation factor are expressed in mouse male germ cells. Proc. Natl. Acad. Sci. U.S.A..

[22] Wallace A.M., Denison T., Attaya E.N., MacDonald C.C. (2004). Developmental differences in expression of two forms of the CstF-64 polyadenylation protein in rat and mouse. Biol. Reprod..

[23] Huber Z., Monarez R.R., Dass B., MacDonald C.C. (2005). The mRNA encoding τCstF-64 is expressed ubiquitously in mouse tissues. Ann. N.Y. Acad. Sci..

[24] Yao C., Choi E.A., Weng L., Xie X., Wan J., Xing Y., Moresco J.J., Tu P.G., Yates J.R., Shi Y. (2013). Overlapping and distinct functions of CstF64 and CstF64τ in mammalian mRNA 3′ processing. RNA.

[25] Dass B., Tardif S., Park J.Y., Tian B., Weitlauf H.M., Hess R.A., Carnes K., Griswold M.D., Small C.L., MacDonald C.C. (2007). Loss of polyadenylation protein τCstF-64 causes spermatogenic defects and male infertility. Proc. Natl. Acad. Sci. U.S.A..

[26] Hockert K.J., Martincic K., Mendis-Handagama S.M.L.C., Borghesi L.A., Milcarek C., Dass B., MacDonald C.C. (2011). Spermatogenetic but not immunological defects in mice lacking the τCstF-64 polyadenylation protein. J. Reprod. Immunol..

[27] Tardif S., Akrofi A., Dass B., Hardy D.M., MacDonald C.C. (2010). Infertility with impaired zona pellucida adhesion of spermatozoa from mice lacking τCstF-64. Biol Reprod..

[28] Ji Z., Lee J.Y., Pan Z., Jiang B., Tian B. (2009). Progressive lengthening of 3’ untranslated regions of mRNAs by alternative polyadenylation during mouse embryonic development. Proc. Natl. Acad. Sci. U.S.A..

[29] Ji Z., Tian B. (2009). Reprogramming of 3’ untranslated regions of mRNAs by alternative polyadenylation in generation of pluripotent stem cells from different cell types. PLoS One.

[30] Hansen G.M., Markesich D.C., Burnett M.B., Zhu Q., Dionne K.M., Richter L.J., Finnell R.H., Sands A.T., Zambrowicz B.P., Abuin A. (2008). Large-scale gene trapping in C57BL/6N mouse embryonic stem cells. Genome Res..

[31] Youngblood B.A., Alfano R., Pettit S.C., Zhang D., Dallmann H.G., Huang N., Macdonald C.C. (2014). Application of recombinant human leukemia inhibitory factor (LIF) produced in rice (Oryza sativa L.) for maintenance of mouse embryonic stem cells. J. Biotechnol..

[32] Kapur N., Mignery G.A., Banach K. (2007). Cell cycle-dependent calcium oscillations in mouse embryonic stem cells. Am. J. Physiol. Cell Physiol..

[33] Livak K.J., Schmittgen T.D. (2001). Analysis of relative gene expression data using real-time quantitative PCR and the 2^−ΔΔCt^ Method. Methods.

[34] Martin G., Gruber A.R., Keller W., Zavolan M. (2012). Genome-wide analysis of pre-mRNA 3′ end processing reveals a decisive role of human cleavage factor I in the regulation of 3′ UTR length. Cell Rep..

[35] Ruepp M.D., Schweingruber C., Kleinschmidt N., Schümperli D. (2011). Interactions of CstF-64, CstF-77, and symplekin: implications on localisation and function. Mol. Biol. Cell.

[36] Yao C., Biesinger J., Wan J., Weng L., Xing Y., Xie X., Shi Y. (2012). Transcriptome-wide analyses of CstF64-RNA interactions in global regulation of mRNA alternative polyadenylation. Proc. Natl. Acad. Sci. U.S.A..

[37] Pease S., Braghetta P., Gearing D., Grail D., Williams R.L. (1990). Isolation of embryonic stem (ES) cells in media supplemented with recombinant leukemia inhibitory factor (LIF). Dev. Biol..

[38] Martincic K., Campbell R., Edwalds-Gilbert G., Souan L., Lotze M.T., Milcarek C. (1998). Increase in the 64-kDa subunit of the polyadenylation/cleavage stimulatory factor during the G0 to S phase transition. Proc. Natl. Acad. Sci. U.S.A..

[39] Takagaki Y., Manley J.L. (1998). Levels of polyadenylation factor CstF-64 control IgM heavy chain mRNA accumulation and other events associated with B cell differentiation. Mol. Cell.

[40] Wagner E.J., Marzluff W.F. (2006). ZFP100, a component of the active U7 snRNP limiting for histone pre-mRNA processing, is required for entry into S phase. Mol. Cell. Biol..

[41] Becker K.A., Ghule P.N., Therrien J.A., Lian J.B., Stein J.L., van Wijnen A.J., Stein G.S. (2006). Self-renewal of human embryonic stem cells is supported by a shortened G1 cell cycle phase. J. Cell. Physiol..

[42] Stein G.S., van Wijnen A.J., Stein J.L., Lian J.B., Montecino M., Zaidi S.K., Braastad C. (2006). An architectural perspective of cell-cycle control at the G1/S phase cell-cycle transition. J. Cell. Physiol..

[43] Hatton L.S., Eloranta J.J., Figueiredo L.M., Takagaki Y., Manley J.L., O'Hare K. (2000). The Drosophila homologue of the 64 kDa subunit of cleavage stimulation factor interacts with the 77 kDa subunit encoded by the suppressor of forked gene. Nucleic Acids Res..

[44] Minvielle-Sebastia L., Winsor B., Bonneaud N., Lacroute F. (1991). Mutations in the yeast RNA14 and RNA15 genes result in an abnormal mRNA decay rate; sequence analysis reveals an RNA-binding domain in the RNA15 protein. Mol. Cell. Biol..

[45] MacDonald C.C., McMahon K.W. (2010). Tissue-specific mechanisms of alternative polyadenylation: testis, brain and beyond. WIREs RNA.

[46] Li W., Yeh H.J., Shankarling G.S., Ji Z., Tian B., MacDonald C.C. (2012). The tauCstF-64 polyadenylation protein controls genome expression in testis. PLoS One.

[47] Narita T., Yung T.M., Yamamoto J., Tsuboi Y., Tanabe H., Tanaka K., Yamaguchi Y., Handa H. (2007). NELF interacts with CBC and participates in 3′ end processing of replication-dependent histone mRNAs. Mol. Cell.

[48] Pirngruber J., Shchebet A., Schreiber L., Shema E., Minsky N., Chapman R.D., Eick D., Aylon Y., Oren M., Johnsen S.A. (2009). CDK9 directs H2B monoubiquitination and controls replication-dependent histone mRNA 3’-end processing. EMBO Rep..

[49] Sullivan K.D., Mullen T.E., Marzluff W.F., Wagner E.J. (2009). Knockdown of SLBP results in nuclear retention of histone mRNA. RNA.

[50] Ideue T., Adachi S., Naganuma T., Tanigawa A., Natsume T., Hirose T. (2012). U7 small nuclear ribonucleoprotein represses histone gene transcription in cell cycle-arrested cells. Proc. Natl. Acad. Sci. U.S.A..

[51] Mannironi C., Erba E., Corda D., Gianellini L., D'Incalci M. (1989). Changes in the synthesis of histone H1(0) and H1 in rat FRTL-5 thyroid cells exposed to thyrotropin. Life Sci..

[52] Akhmanova A., Miedema K., Wang Y., van Bruggen M., Berden J.H., Moudrianakis E.N., Hennig W. (1997). The localization of histone H3.3 in germ line chromatin of Drosophila males as established with a histone H3.3-specific antiserum. Chromosoma.

[53] Lanzotti D.J., Kaygun H., Yang X., Duronio R.J., Marzluff W.F. (2002). Developmental control of histone mRNA and dSLBP synthesis during Drosophila embryogenesis and the role of dSLBP in histone mRNA 3’ end processing in vivo. Mol. Cell. Biol..

[54] Moss S.B., Ferry R.A., Groudine M. (1994). An alternative pathway of histone mRNA 3’ end formation in mouse round spermatids. Nucleic Acids Res..

[55] Dominski Z., Marzluff W.F. (1999). Formation of the 3’ end of histone mRNA. Gene.

[56] Fahrner K., Yarger J., Hereford L. (1980). Yeast histone mRNA is polyadenylated. Nucleic Acids Res..

[57] Davila Lopez M., Samuelsson T. (2008). Early evolution of histone mRNA 3’ end processing. RNA.

[58] Marzluff W.F., Duronio R.J. (2002). Histone mRNA expression: multiple levels of cell cycle regulation and important developmental consequences. Curr. Opin. Cell Biol..

