# Supplementary material for: CstF-64 supports pluripotency and regulates cell cycle progression in embryonic stem cells through histone 3′ end processing
Source: Nucleic Acids Res. 2014 Jun 21;42(13):8330–42. doi: 10.1093/nar/gku551 (PMC4117776; doi:10.1093/nar/gku551)
Supplement: SUPPLEMENTARY DATA [file supp_42_13_8330__index.html]

CstF-64 supports pluripotency and regulates cell cycle progression in embryonic stem cells through histone 3′ end processing — SUPPLEMENTARY DATA 

# CstF-64 supports pluripotency and regulates cell cycle progression in embryonic stem cells through histone 3′ end processing

## SUPPLEMENTARY DATA

**Files in this Data Supplement:**

- SUPPLEMENTARY DATA
- SUPPLEMENTARY DATA
- SUPPLEMENTARY DATA
- SUPPLEMENTARY DATA
- SUPPLEMENTARY DATA
- SUPPLEMENTARY DATA
