# Supplementary figures and images for: CstF-64 supports pluripotency and regulates cell cycle progression in embryonic stem cells through histone 3′ end processing
Source: Nucleic Acids Res. 2014 Jun 21;42(13):8330–42. doi: 10.1093/nar/gku551 (PMC4117776; doi:10.1093/nar/gku551)

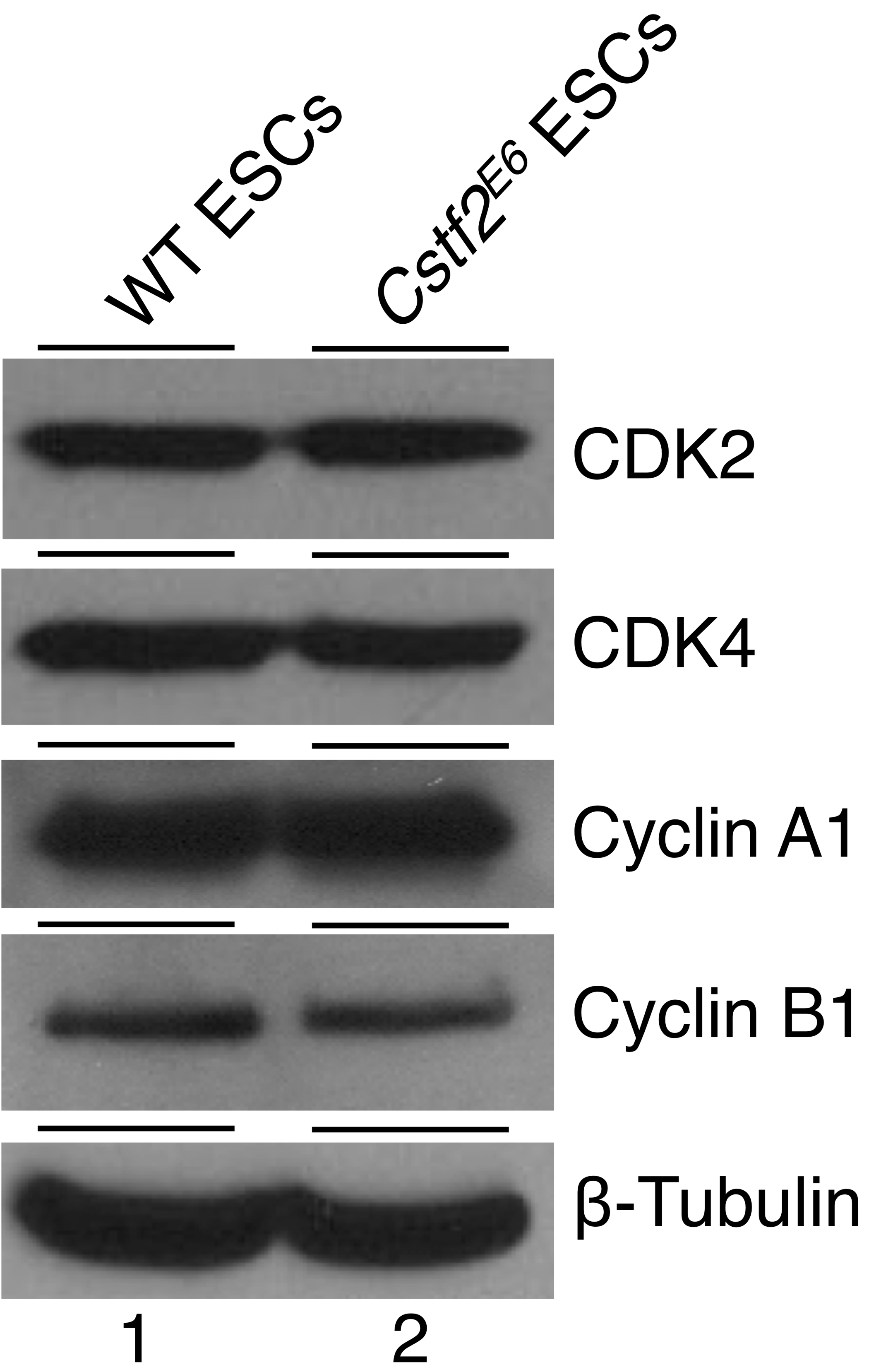

Supplement: SUPPLEMENTARY DATA [file supp_gku551_nar-01051-v-2014-File009.tif]

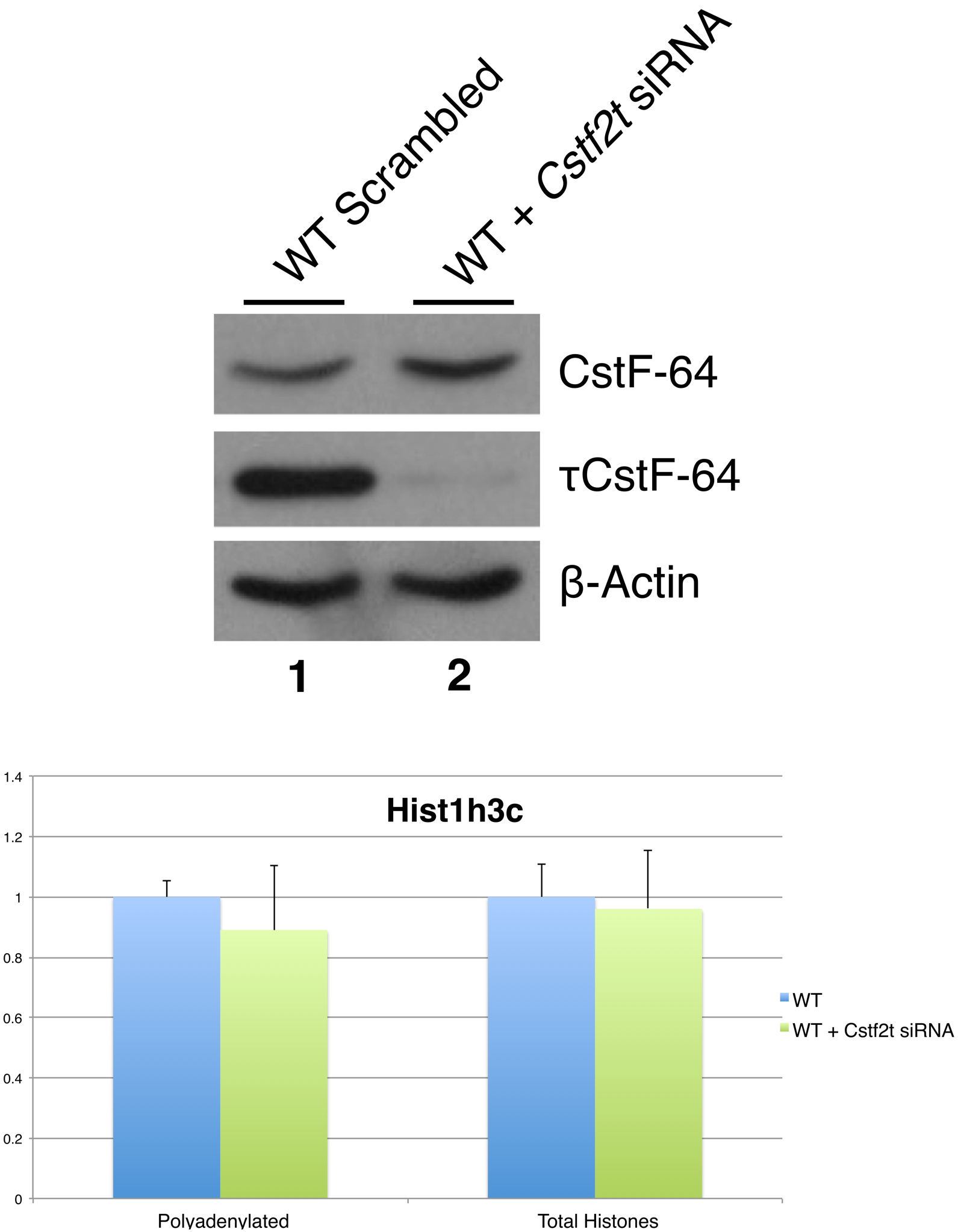

Supplement: SUPPLEMENTARY DATA [file supp_gku551_nar-01051-v-2014-File011.tif]
