# Supplementary material for: CstF-64 supports pluripotency and regulates cell cycle progression in embryonic stem cells through histone 3′ end processing
Source: Nucleic Acids Res. 2014 Jun 21;42(13):8330–42. doi: 10.1093/nar/gku551 (PMC4117776; doi:10.1093/nar/gku551)
Supplement: SUPPLEMENTARY DATA [file supp_gku551_nar-01051-v-2014-File010.docx]

**Supplementary Table 3. Cleavage and polyadenylation sites**

************ *****

*Hist1h2aa* aaaGGCUCUUUUCAGAGCCaccuucauuuucacgaaaagcugugcaugac

*Hist1h2ab* aaaGGCUCUUUUCAGAGCCacccacaucuuuccauaaaaugagcugccac

*Hist1h2ac* aaaGGCUCUUUUCAGAGCCcccacuuuuucaaaagaagaguugaacagga

*Hist1h2ae* aaaGGCUCUUUUCAGAGCCauucacuuaauucaguaaagugcugaaauac

*Hist1h2af* aaaGGCUCUUUUCAGAGCCaccacuucuucauauaagagucuaguaacAU

*Hist1h2ag* aaaGGCUCUUUUCAGAGCCaccuaaccuuuccuaacaagggcuuagcacu

*Hist1h2ah* aaaGGCUCUUUUCAGAGCCacacacaaaguccagaaagaagcugguacau

*Hist1h2ai* aaaGGCUCUUUUCAGAGCCacucacuuucuccaaagagaccuaaacacua

*Hist1h2ak* aaaGGCUCUUUUCAGAGCCaccuacaaaaacacuuggaggaguugugcua

*Hist1h2an* aaaGGCUCUUUUCAGAGCUaacacauuugucuuuuuaaggagcuuaagcu

*Hist1h2ao* aaaGGCUCUUUUCAGAGCCauccacagugucuuuuaaagaacugaauacu

*Hist2h2ab* aaaGGCUCUUUUAAGAGCCaccaaagugucauccaaagggcuggucacaa

*Hist2h2ac* aaaGGCUCUUUUUAGAGCCaccaaaucuuuuacaaaggagcuagcuaacg

*Hist2h2aa1* aaaGGCUCUUUUCAGAGCCacccacugaaucagauaaagaguugugucac

*Hist3h2a* aaaGGCUCUUUUCAGAGCCacccacaaccucauuagaaagcgcuguacac

**** *******

*Hist1h2ba* aaaGGCUCUUUUAAGAGCCacccaccucuucuguugagagagcugcguuu

*Hist1h2bb* aaaGGCUCUUUUAAGAGCCacccacacuuuucagaaaagaaguuguaaua

*Hist1h2be* aaaGGCUCUUUUCAGAGCCacccaacaguucaaaauugagcuaugcgcuu

*Hist1h2bf* aaaGGCUCUUUUCAGAGCCaccuacccuuucaaaaagggagcuguugcca

*Hist1h2bg* aaaGGCUCUUUUCAGAGCCacccauacugucacuuuaagugcuuuguaau

*Hist1h2bh* aaaGGCUCUUUUCAGAGCCacucaagacuucaaaauuggagcuuuaaugc

*Hist1h2bj* aaaGGCUCUUUUCAGAGCCacgcacaauuuuuaaagagagcugcugcaug

*Hist1h2bk* aaaGGCUCUUUUAAGAGCCacuaaaccaccaguauuagagcugcaaacau

*Hist1h2bl* aaaGGCUCUUUUAAGAGCCacccacacuuuccucuggaaaagcuguugca

*Hist1h2bm* aaaGGCUCUUUUCAGAGCCacuaagcaguucuuugaaaagggcuagcaca

*Hist1h2bn* AAAGGCUCUUUUCAGAGCCACCCACACUUUCAacUaaaaaaggcUgUUgc

*Hist2h2bb* aaaGGCUCUUUUCAGAGCCacuuuaguucucaaaaaaagacuguaagcac

*Hist2h2be* aaaGUCUCUUUUAGUGAGUcacccgcucaaaucagaagagagcuuugcca

*Hist3h2ba* aaaGGCUCUUUUCAGAGCCaccuuccaagcucaaaaaaggagcgugcuac

*Hist3h2bb* aaaGGCUCUUUUCAGAGCCacccauacagucguuaaaagggucuugaaca

***** **** * *** **

*Hist1h4a* aaaGGCCCUUCUCAGGGCCgcccacaUUcccUgaaaaagcgcUgUaaUac

*Hist1h4b* aaaGGUCCUUUUCAGGACCacUcacUgaaUUccUUaaaaagcUgUgcaUU

*Hist1h4c* aaaGGCCCUUUUCAGGGCCacccacaaaUUccUaggagUUgUUcacUUac

*Hist1h4d* aaaGGCCCUUUUCAGGGCCacUaccUUcUcacUUaaaggagcUgcUcUUa

*Hist1h4f* aaaGGCCCUUUUCAGGGCCacccacUUUgUcaUaaaaagcUgUUUgcaUU

*Hist1h4h* aaaGGCCCUUUUCAGGGCCgcccacaUUUUcUUaUUaaaagagcgaacaU

*Hist1h4i* aaaGGCCCUUUUUAGGGCCaccacaaaaUcaaaagagcUaagUUgcUUUU

*Hist1h4j* aaaGGUCCUUUUCAGGACCaccacaUcUUUUacagaagagcUgacgcUUg

*Hist1h4m* aaaGGCCCUUUUCAGGGCCaUccaaUUaaUccUccaaagggUUgcaacaU

*Hist1h4k* aaaGGCCCUUUUCAGGGCCaUccaaUUaaUccUccaaagggUUgcaacaU

*Hist2h4* aaaGGCCCUUUUCAGGGCCcacaaagcaUcagaaaggagcUgUggacaUU

*Hist2h4* aacGGCCCUUUUUAGGGCCaaccacagUcUcUUcaggagagcUgacacUg

Underlined nucleotides are the one different from the consensus sequence in the stem-loop region of the histone mRNAs

Highlighted in yellow is the stem region of the stem-loop structure.

In light blue color is the loop region of the stem-loop

Nucleotides in red are the one that we observed cleavage and polyadenylation using the A-seq technique in wild type embryonic stem cells and *Cstf2^E6^* depleted embryonic stem cells.

In the green color is the HDE sequence (consensus: AAAGAGCUGU)

Asterisk at the top of each alignment indicates the identical nucleotides in all of the given histone family members.

## Supplementary Table 4. Primers used in the study

| **Target** | **Forward (5'-3')** |
| --- | --- |
|  | **Reverse (5'-3')** |
| Oct4 (*Pou5f1*) | GCCGTCTTTCCACCAGGCCC |
|  | TCGAGGATCCACCCAGCCCG |
| *Nanog* | CCTGGTCCCCACAGTTTGCCT |
|  | GCAGGTCTTCAGAGGAAGGGCG |
| *Klf4* | GAAGGTCGTGGCCCCGGAAA |
|  | CCGGTGCCCTGTGTGTTTGC |
| *Lefty2* | CTCAGATGGGGCGCTCATAC |
|  | AGCAAAGGTCTGACGAGAGC |
| Brachyury (*T*) | CTGCCAGCAGCCGGGGTATTC |
|  | GGCTGGGGTAGGGCGATGAC |
| *Nkx6-3* | TTTGGGAGCATCTCAGCAGG |
|  | GGGGACGGTTTCTGGGATTT |
| *Olig2* | TACAGACCGAGCCAACACCA |
|  | GGCCCCAGGGATGATCTAAG |
| *Cstf2* | GGCCCTGGTTCCTTAGCCCCT |
|  | GCAAGGCTCCCCGCTGCATA |
| *Cstf2t* | CAACCGTGGCCCTGCCTCAC |
|  | GGGCCGAGTCTCCATCCCTCG |
| H3 | CTCCTCCCAAGAATGGCTCG |
|  | CTCGGTCGACTTCTGGTAGC |
| T7 U7 snRNA | AAGTGTTACAGCTCTTTTAG |
|  | GGTAATACGACTCACTATAGGGGGGTTTTTCCGACCGAAGTCA |
| T7 H3 | CTCCTCCCAAGAATGGCTCG |
|  | GGTAATACGACTCACTATAGGGCATGATGGTGACACGCTTGG |

## Supplementary Table 5. Quality control of RNA-seq data

| Sample name | Clean reads | Clean bases | Q20(%) | GC content(%) |
| --- | --- | --- | --- | --- |
| RNA-seq WT-ESC-FF2 | 41,270,964 | 3,714,386,760 | 97.08 | 48.23 |
| RNA-seq *Cstf2^E6^*-FF2 | 41,537,230 | 3,629,215,673 | 97.08 | 48.44 |
| RNA-seq WT-ESC-FF3 | 62,838,304 | 5,655,447,360 | 97.43 | 49.88 |
| RNA-seq *Cstf2^E6^*-FF3 | 53,439,826 | 4,809,584,340 | 97.49 | 49.76 |
