# Supplementary material for: CstF-64 supports pluripotency and regulates cell cycle progression in embryonic stem cells through histone 3′ end processing
Source: Nucleic Acids Res. 2014 Jun 21;42(13):8330–42. doi: 10.1093/nar/gku551 (PMC4117776; doi:10.1093/nar/gku551)
Supplement: SUPPLEMENTARY DATA [file supp_gku551_nar-01051-v-2014-File014.docx]

**CstF-64 Supports Pluripotency and Regulates Cell Cycle Progression in Embryonic Stem Cells through Histone 3′ End Processing**

Bradford A. Youngblood, Petar N. Grozdanov and Clinton C. MacDonald

Department of Cell Biology & Biochemistry, Texas Tech University Health Sciences Center, 3601 4th Street, Lubbock, Texas 79430-6540, USA

# Supplementary Figure Legends

**Supplementary Figure 1:** Expression of G_1_/S and G_2_/M phase regulators CDK2, CDK4, Cyclin A1, and Cyclin B1 do not change in *Cstf2^E6^* cells. Shown are Western blots of CDK2, CDK4, Cyclin A1, and Cyclin B1 in wild type ESCs (lanes 1) and *Cstf2^E6^* cells (lanes 2).

**Supplementary Figure 2:** siRNA-mediated knockdown of *Cstf2t* does not alter polyadenylation of *Hist1h3c* in wild type mouse ESCs. (Top) τCstF-64 expression was reduced by transfection with a *Cstf2t*-specific siRNA in wild type mouse ESCs (lanes 2). (Bottom) Neither oligo(dT)-primed (Polyadenylated) nor random-primed (Total Histones) *His1h3c* mRNA levels changed significantly upon knockdown of *Cstf2t* in wild type mouse ESCs.
